# Formation of optical supramolecular structures in a fibre laser by tailoring long-range soliton interactions

**DOI:** 10.1038/s41467-019-13746-6

**Published:** 2019-12-17

**Authors:** W. He, M. Pang, D. H. Yeh, J. Huang, C. R. Menyuk, P. St. J. Russell

**Affiliations:** 1 Max Planck Institute for the Science of Light, Friedrich Alexander University, Staudtstrasse 2, 91058 Erlangen, Germany; 2 Department of Computer Science and Electrical Engineering, University of Maryland Baltimore County, Baltimore, MD 21250 USA; 3 Department of Physics, Friedrich Alexander University, Staudtstrasse 2, 91058 Erlangen, Germany; 4 Present Address: State Key Laboratory of High Field Laser Physics, Shanghai Institute of Optics and Fine Mechanics, Chinese Academy of Sciences, 201800 Shanghai, China

**Keywords:** Fibre lasers, Mode-locked lasers, Nonlinear optics, Solitons

## Abstract

Self-assembly of fundamental elements through weak, long-range interactions plays a central role in both supramolecular DNA assembly and bottom-up synthesis of nanostructures. Optical solitons, analogous in many ways to particles, arise from the balance between nonlinearity and dispersion and have been studied in numerous optical systems. Although both short- and long-range interactions between optical solitons have attracted extensive interest for decades, stable soliton supramolecules, with multiple aspects of complexity and flexibility, have thus far escaped experimental observation due to the absence of techniques for enhancing and controlling the long-range inter-soliton forces. Here we report that long-range soliton interactions originating from optoacoustic effects and dispersive-wave radiations can be precisely tailored in a fibre laser cavity, enabling self-assembly of large numbers of optical solitons into highly-ordered supramolecular structures. We demonstrate several features of such optical structures, highlighting their potential applications in optical information storage and ultrafast laser-field manipulation.

## Abstract (web summary)

Optical solitons have been studied in a variety of systems for their unique properties. Here, the authors report on optically observed solitonic supramolecules, made up of large-scale structures of many solitons interacting within a fiber cavity, and study their fundamental characteristics.

## Introduction

Optical solitons, arising from a stable balance between nonlinear and dispersive effects, are often regarded as ideal fundamental elements in fibre-optic telecommunication systems^1,2^, optical information storage^3–6^, and optical signal processing^7^ due to their intrinsic self-localization. Analogous in many ways to particles, both spatial and temporal solitons have been demonstrated in semiconductor lasers^8,9^, passive fibre loops^4,10,11^, optical microresonators^12^, and fibre lasers^13^. Precise control and long-term stabilization of multiple-soliton patterns in these systems are the key techniques for storage and transmission of optical information and manipulation of ultrafast laser fields^5,9–11,13^. While temporal tweezing of optical solitons using external modulation provides an elegant means of manipulating optical solitons in passive fibre loops^10^ or semiconductor lasers^14^, the formation of self-stabilized light structures in a variety of optical systems through short-range^13,15–18^ and long-range^5,19–21^ interactions between optical solitons has recently attracted considerable interest.

Multiple optical solitons, propagating together in close proximity, can strongly interact through their tailing fields, resulting in the formation of robust, phase-locked bound states that are frequently referred as soliton molecules^15–17,22^, soliton macromolecules^13,23^, or soliton crystals^18,24^, in analogy to their chemical counterparts that are formed by strong covalent bonds. In practice, such strong, short-range interactions usually result in narrow spacing between adjacent solitons, severely challenging real-time characterization of their detailed profiles^22^. Long-range interactions between solitons can lead to soliton binding with internal spacings tens to hundreds of times greater than the duration of the individual solitons, and several mechanisms for such long-range soliton interactions have been extensively studied, mediated by Casimir-like^21,25^, perturbation-induced^26–28^, thermal effects^29^, and optoacoustic effects^5,19,28^. However, long-range soliton interactions in previous systems are generally ultra-weak and difficult to control, resulting in transient inter-solitonic forces^19,21,28,29^, harmonically mode-locked laser pulses with erratic repetition rates and high timing jitter^28,30,31^, or limited numbers of weakly-bound solitons^20,21^. The difficulty in forming stable, macroscopic solitonic structures is mainly due to the absence of effective approaches to enhancing and controlling long-range inter-soliton interactions. We recently reported that intense optomechanical effects in a short length of solid-core photonic crystal fibre (PCF) could be used to form a robust, GHz-rate optomechanical lattice in a soliton fibre laser^32–34^ and that individual solitons could be selectively erased by launching precisely timed erasing pulses^5^.

In this paper we report that long-range forces of different physical origin between a variety of solitonic elements can be widely tailored to cooperate with each other in a fibre laser cavity, leading to the self-assembly of a large population of optical solitons into highly-ordered, supramolecular structures^35,36^. These structures are found to exhibit unprecedented complexity in their configuration, multiple degrees of freedom, while at the same time featuring long-term stability, elementary diversity, structural flexibility, and reversibility.

## Results

### Illustration of concept

**Fig. 1:**
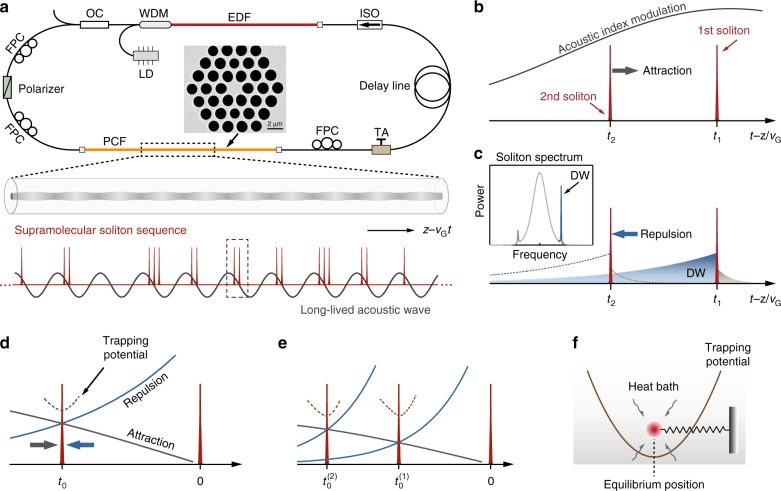
Conceptual illustration of a supramolecular assembly of optical solitons in a fibre laser cavity. **a** Sketch of the experimental set-up; the inset shows a scanning electron micrograph (SEM) of the photonic crystal fibre microstructure. The supramolecular soliton sequence propagating in this fibre laser cavity drives an acoustic resonance in the PCF core, creating an optomechanical lattice. Each unit of the optomechanical lattice can accommodate multiple solitons. EDF erbium-doped fibre, WDM wavelength-division multiplexer, LD laser diode, OC output coupler, FPC fibre polarization controller, TA tunable attenuator, ISO isolator. **b** Within each unit of the optomechanical lattice, a long-range optomechanical force of attraction arises between the solitons. **c** A competing force of repulsion appears due to dispersive wave perturbations. The inset shows a typical soliton spectrum with two Kelly sidebands of unequal intensities. **d** Competition between these two long-range forces forms a temporal potential, trapping the second soliton. **e** Stable multi-soliton units can form through the cascaded build-up of trapping potentials. **f** The timing jitter of an individual soliton in a supramolecule is analogous to the thermal motion of a single particle trapped in a harmonic potential.

The mode-locked fibre laser loop that we build to study soliton supramolecules is sketched in Fig. 1a (see details plotted in Supplementary Fig. 1 and described in Supplementary Note 1). A 2-m-long solid-core silica photonic crystal fibre (PCF) with a GHz-rate acoustic core resonance^37^ is inserted into the laser cavity. Upon increasing the pump power of the Er-doped fibre amplifier (EDFA), a variety of stable supramolecular structures composed of a large population of optical solitons can be generated in the laser cavity, all the solitons involved in such structures being globally ordered by optomechanical interactions^5,33,34^. A long-lived acoustic wave in the PCF-core is driven coherently by the soliton sequence, and acts back on the pulses, linking them together by modulating their carrier frequencies, and forming a temporal optomechanical lattice with a period equal to a cycle of acoustic vibration^5^. This global lattice divides the laser cavity into many time-slots of identical length, each of which can accommodate multiple solitons (see Fig. 1a).

Long-range binding of multiple solitons in each optomechanical unit originates from the balance between attractive and repulsive inter-soliton forces. As the solitons ride on the acoustic wave whose wavefront (phase) velocity equals the group velocity of the soliton sequence^5^, the index modulation caused by the acoustic wave leads to a shift in the soliton carrier frequency as the soliton propagates (see Fig. 1b). The magnitude of this frequency shift is determined by the slope of the underlying index modulation^38^. Since the two solitons within one acoustic period are located at different positions (see Fig. 1b), their frequencies shift at rates that differ slightly from each other. The divergence in soliton frequencies, acting in concert with the group-velocity dispersion of the optical fibre, leads to an effective force of attraction. As illustrated in Fig. 1c, the first higher-frequency dispersive wave sideband^39,40^, shed from the first soliton, propagates faster than the soliton and eventually reaches the second soliton, perturbing it through cross-phase modulation^41^. Such dispersive wave perturbations effectively create a repulsive force between the two solitons^27,28^, balancing the force of attraction due to the optoacoustic effect (see theoretical modelling in Supplementary Note 2 with Supplementary Figs. 2 and 3). In principle, more than two solitons can be stably bound within one unit through the cascaded balance of long-range forces (see Fig. 1a, e).

The balance of these two long-range forces results in stable soliton spacing, effectively creating inter-solitonic springs that trap the solitons at each equilibrium position. As a result, noise-induced fluctuations in soliton spacing (timing jitter) occur that are analogous to the thermal motion of a particle trapped in a harmonic potential (Fig. 1g). These can be modelled using a Langevin equation^42^ (see Supplementary Note 3). When pulses are trapped in a harmonic potential, the timing jitter does not grow with time, even though the heat bath (noise source) continuously disturbs the system^42,43^ (see Supplementary Figs. 4 and 5).

### Enabling techniques

Stable soliton supramolecules can be generated experimentally only by carefully designing the fibre laser cavity so that the evolution of long-range forces between hundreds of solitons is precisely controlled. Optoacoustic effects in conventional single-mode fibre are ultra-weak^28,37^, generally leading to harmonically mode-locked pulse trains with high noise levels^28,30,31^. In our laser cavity, in contrast, tight confinement of both optical and acoustic waves in the 1.95-μm-diameter PCF core leads to the enhancement of acoustic-wave-mediated inter-soliton forces by more than two orders of magnitude^5,32^. The excellent robustness of the resulting temporal optomechanical lattice makes possible manipulation of the fine structure within each time-slot of the lattice^5^. In practice, tuning the acoustic-wave amplitude in the PCF core can be realised by detuning of the pulse repetition rate from the acoustic resonant frequency^32,33^, which itself can be adjusted by fabricating PCFs with different core diameters^32,37^.

On the other hand, dispersive-wave generation in soliton fibre lasers, leading to uncontrolled disturbance to the pulse train^6,26–28^, is widely regarded as undesirable, rather than as a source of exploitable inter-solitonic forces. The idea reported here is to stably balance long-range dispersive-wave and optoacoustic effects. In our laser cavity the strength of the dispersive waves shed by individual solitons, which determines the strength of repulsive inter-soliton forces (see Supplementary Note 2), must be tailored so as to counterbalance the attractive inter-soliton forces due to the strong optoacoustic effects. We reveal in the experiments that careful management of both cavity dispersion^44^ and cavity loss are of great importance in determining the strengths and directions of the inter-soliton forces due to dispersive-wave radiation (see Methods).

### Supramolecules with single solitons as building blocks

**Fig. 2:**
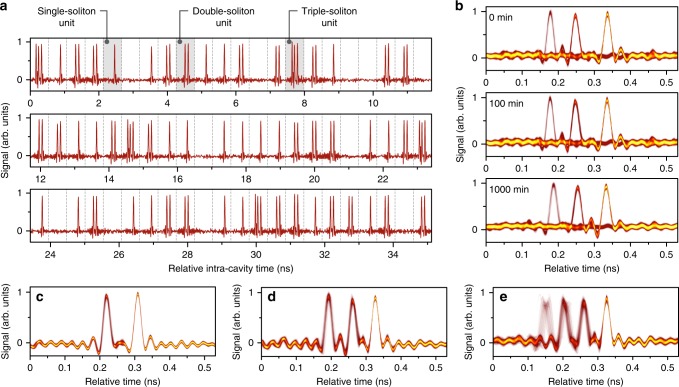
Typical soliton supramolecules and their long-term stability. **a** Time-domain trace of a typical soliton supramolecule (only 66 out of 154 time-slots are shown). **b** Persistence-mode recordings of this soliton supramolecule at 0, 100, and 1000 min. **c–e** Persistence-mode recordings of three typical soliton supramolecules containing two solitons in every unit (**c**), three solitons in every unit (**d**), and both triple- and quadruple-soliton units (**e**).

A typical soliton supramolecule with a chain of units, each containing 0, 1, 2, or 3 trapped solitons, is recorded using a fast detector and an oscilloscope. The resulting time-domain trace is shown in Fig. 2a, where the underlying grid is globally locked to the 1.887 GHz acoustic resonance in the PCF core (period ~532 ps). The duration of individual solitons is measured to be 650 fs. This self-assembled solitonic structure, once formed, is robust, the pulse spacings in each unit being 80 ps between the first and second solitons and 70 ps between the second and third. Monitoring the soliton supramolecule over 1000 min (see Fig. 2b) reveals no measurable degradation in signal-to-noise ratio. This time interval corresponds to stable propagation over 12 billion kilometers (~84 astronomical units) in the freely-running fibre laser loop (see Supplementary Note 4 and Supplementary Figs. 6 for experimental details). The estimated pulse timing jitter is always below 5 ps, measured using the oscilloscope in persistence-mode (see Fig. 2b). In order to monitor the carrier-wave phase of the solitons, we use a narrow-linewidth local oscillator in the form of a single-frequency fibre laser to heterodyne with the supramolecular soliton sequence^33^. The results reveal that the carrier-wave phases of assembled solitons are uncorrelated (see Supplementary Note 5 and Supplementary Figs. 7 for experimental details); in this respect, soliton supramolecules self-assembled through long-range forces differ from soliton molecules strongly bound by short-range forces^22^, when the carrier-waves are phase coherent.

In the experiments, we discover that the system tends to evolve into a supramolecular structure with roughly even distributions of optical solitons, although, since the structure directly emerges from noise by self-organization, the exact number of solitons differs randomly from time-slot to time-slot. Through careful adjusting both the laser pump power and intra-cavity polarization controllers, we can partially control the fine structure of the self-assembled soliton supramolecule, reproducibly generating structures in which every time-slot contained the same number of soliton units (single^5,33,34^, double, or triple) as shown in Fig. 2c, d. We can also generate a supramolecular soliton stream containing both triple and quadruple soliton units (see Fig. 2e). More structural details of these supramolecules with single solitons as building blocks are described in Supplementary Notes 6 and 7 with additional experimental illustrations in Supplementary Figs. 8–11. Although so far we have not demonstrated independent control of individual solitons in the supramolecular structure, fast encoding of the supramolecular patterns should be possible by launching a timed sequence of writing pulses into the laser cavity or modulating the pump laser power^5,9,11^.

### Elementary diversity

**Fig. 3:**
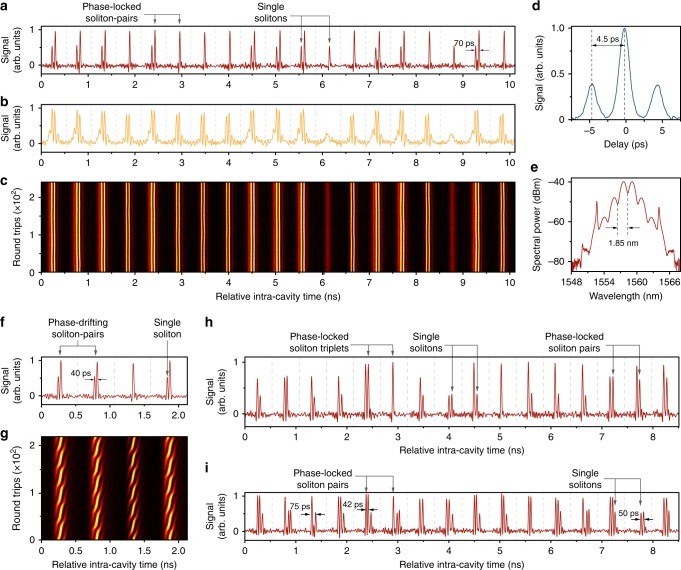
Elementary diversity of the soliton supramolecules. **a**–**c** The time-domain trace (**a**) and TS-DFT signal (**b**) of a soliton supramolecule with both single solitons and soliton pairs as building blocks (only 19 out of 154 time-slots are shown). The persistence-mode plot in **c** confirms that the inner spacing and phase differences in the soliton pairs remain constant during the measurement. **d**, **e** Autocorrelation trace and optical spectrum of the soliton supramolecule. **f, g** Time-domain trace (**f**) and TS-DFT signal (**g**) of a soliton supramolecule with phase-sweeping soliton-pairs as one of its building blocks (only 4 out of 154 time-slots are shown). **h** Time-domain trace of a soliton supramolecule comprising single solitons, soliton pairs, and soliton triplets. **i** This complex supramolecule illustrates how the varying forces between dissimilar building blocks can lead to different inter-soliton spacings. Only 16 out of 154 time-slots are shown in 3e and 3 f.

The elementary diversity of the supramolecular structures can be greatly increased by incorporating additional fundamental building blocks. For example, both single solitons and phase-locked soliton pairs^15–17^ can be incorporated as building blocks in the supramolecular structure, as seen in the time-domain trace in Fig. 3a–c. This type of supramolecular structure is held together by both long-range and short-range inter-soliton interactions, in a manner reminiscent of atoms in biochemical supramolecules^36^, which self-assemble through a combination of short-range and long-range forces.

In the recorded time-domain sequence in Fig. 3a–c, the bandwidth (33 GHz) of the oscilloscope is not sufficient to resolve individual solitons in a pair, with the result that the soliton pairs appear as pulses with twice the amplitude of a single soliton. To overcome this limitation, we use a time-stretched dispersive Fourier transform (TS-DFT) to record a real-time interferogram of the soliton sequence^22^. In time-slots with higher amplitude pulses, strong fringes appear, confirming that they contain phase-locked soliton pairs with a short separation. Since many of the building blocks of this soliton supramolecule are soliton pairs with identical spacings and phase differences, second-harmonic autocorrelation can be used to directly measure the temporal spacing of the soliton pairs, which turned out to be Δ*t* ~ 4.5 ps (see Fig. 3d). In addition, using an optical spectral analyser we observe strong spectral interference with a period Δ*λ* = 1.85 nm ~ *λ*^2^/(*c*Δ*t*) (see Fig. 3e). Structural details of such supramolecules are shown in Supplementary Figs. 12 and 13 and described in Supplementary Note 8.

Experimentally we find that more types of elementary building blocks can exist in the assembly, which dramatically increases the complexity of the soliton supramolecule. For example, we observe that phase-drifting soliton pairs^45^ (see Fig. 3f, g) and phased-locked soliton-triplets (see Fig. 3h) can be included in supramolecules. (More details on these different soliton molecules in the supramolecular structures are given in Supplementary Figs. 14–16). Soliton molecules with different inner spacings and phase relations can coexist in the same soliton supramolecule, further increasing the structural complexity (see examples in Supplementary Figs. 17 and 18). Moreover, the spacing between different building blocks in the supramolecule can differ due to varied long-range forces. As shown in Fig. 3i, several characteristic internal spacings (75 ps, 42 ps and 50 ps) are observed in a soliton supramolecule composed of both single solitons and phase-locked soliton pairs, corresponding, respectively, to different interactions of pair-to-one, pair-to-pair, and one-to-one interactions (see Supplementary Fig. 19 for more examples). The diversity of building blocks in the supramolecular structures greatly enriches the range of possible encoding strategies when such structures are used for carrying digital information. Instead of exclusively varying the soliton number in each time-slot, different soliton bound states (long-range or short-range) can be used in the encoding format. These bound states can easily be discriminated using fast detectors, and since the combined optical energy does not vary significantly from time-slot to time-slot, the optomechanical binding forces remain constant, leading to a stable optomechanical lattice.

### Structural flexibility and reversibility

**Fig. 4:**
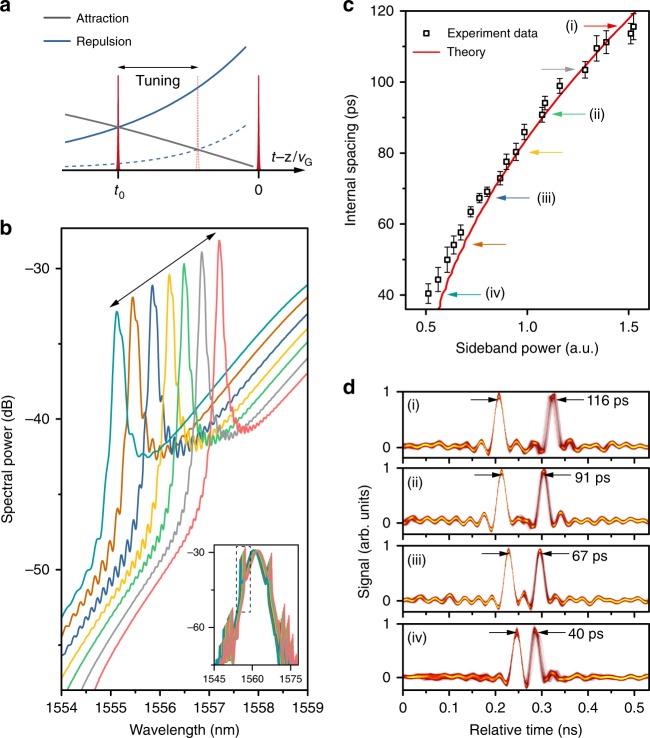
Continuous tuning of the long-range forces between optical solitons. **a** Tailoring the pulse spacing within each unit of the supramolecular structure by varying the long-range, inter-soliton forces. **b** Showing how the spectral intensity in the first higher-frequency sideband decreases when the cavity loss is increased; the inset shows that the overall soliton bandwidth is almost independent of cavity loss. **c** The dependence of the soliton spacing on the side-band intensity. The experimental data-points, plotted as black squares (standard deviations are shown as error bars), agree well with the red theoretical curve (see Supplementary Note 2 and Note 9). **d** Four typical persistence-mode traces of the soliton supramolecule during the spacing tuning process, corresponding to the data-points (i), (ii), (iii), and (iv) in **c**.

The weak nature of the long-range interactions renders the soliton supramolecules highly reconfigurable. For example, their inner structure can change in response to variations in the long-range, inter-soliton forces (see Fig. 4a). We find that the spacing between the long-range bound solitons in one unit of the optomechanical lattice can be continuously tuned over a large range while maintaining the overall supramolecular structure. By placing a tunable attenuator in the laser cavity (Fig. 1a) we are able to adjust the cavity gain and loss, permitting continuous tuning of the dispersive wave intensity. In particular, we are able to double the intensity (Fig. 4b), dramatically reinforcing the repulsive force between the solitons. As shown in Fig. 4c, this leads to an increase in the internal soliton spacing from 40 ps and 116 ps in a supramolecule with two solitons in every time-slot (see experimental details in Supplementary Note 9). Supramolecules with three solitons in every time-slot can also be tuned in the same way (See Supplementary Fig. 20). We can also cycle the soliton spacing back and forth by adjusting the cavity length so as to vary the amplitude of the acoustic wave and thus the attractive force (see Supplementary Fig. 21).

**Fig. 5:**
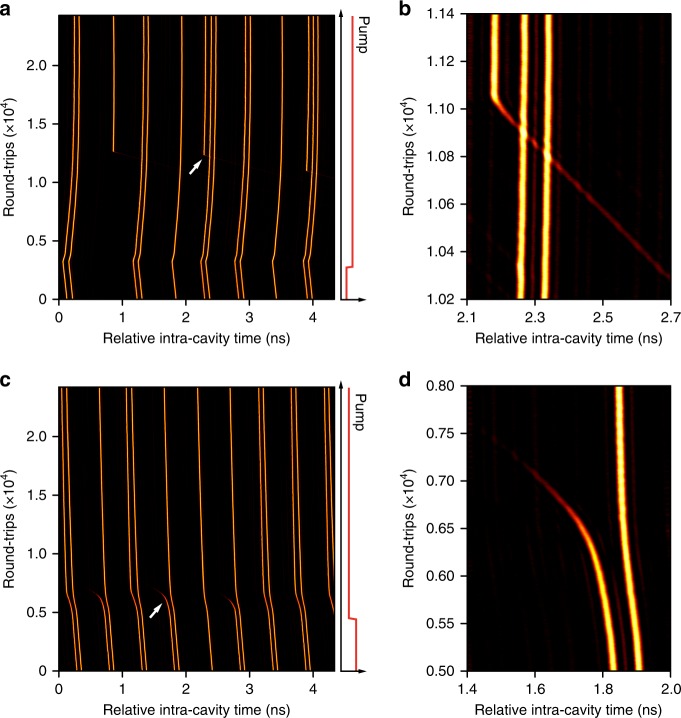
Addition and removal of solitons to and from supramolecular structures. **a**, **b** An abrupt jump in EDFA pump power leads to the addition of solitons to a soliton supramolecule without destroying its basic structure. **c**, **d** Removal of some solitons from a soliton supramolecule can also be achieved by decreasing the pump power.

The supramolecular pattern can switched to a new state by abruptly perturbing the system. Both addition and removal of individual solitons (see Fig. 5) are possible. For example (Fig. 5a), an abrupt increase of ~15% in the EDFA pump power over ~1 µs resulted in the generation of additional solitons (see experiment details described in Supplementary Note 10). The increased pump power leads to higher soliton intensities and thus lower group velocities, as seen in a sudden bending of the pulse trajectories (Fig. 5a), corresponding to variations in the round-trip time. We also find that, as a result of the increased background noise at higher pump power, noisy spikes sometimes turned into stable solitons after a transition period^46^ (Fig. 5a). These newly created solitons are then incorporated into the supramolecule via the cascaded binding mechanism depicted in Fig. 1e, increasing the numbers of solitons in a few time-slots (see the expanded view of a typical capturing process shown in Fig. 5b, corresponding to the region marked by the white arrow in Fig. 5a). In the experiments we can also remove solitons from a supramolecule (Fig. 5c) by decreasing the EDFA pump power by ~10% over ~1 µs, causing many double-soliton units to degrade into single-soliton units (see Fig. 5d for a zoom-in to a typical process of soliton fade-away). Notably, the supramolecular structures that encounter abrupt perturbations in pump power, after experiencing a transient process of self-adjustment (see detailed experimental recordings in Supplementary Figs. 22–25), can quickly settle down to a stable structure, indicating a possible means of fine control (information encoding) of the supramolecular pattern.

## Discussion

Although supramolecules are well-known biological and biochemical structures, they have not previously been observed in an optical setting. Weak long-range inter-soliton interactions make it possible to create solitonic supramolecules in fibre laser cavities passively mode-locked by optoacoustic effects. The temporal spacing between solitons in the supramolecule can be adjusted from tens to hundreds of picoseconds—easily resolvable with fast electronics. In contrast to conventional soliton molecules, which are localized structures composed of a small number of phase-correlated solitons^13,15–17^, supramolecules are large-scale structures, composed of a large number of solitons and tightly-bound soliton molecules, distributed over the entire fibre laser cavity with a built-in hierarchy.

Biochemical and biological supramolecules^35,36^ have many features in common with these newly-discovered optical counterparts, as well as important differences. On one hand, biological and biochemical supramolecular structures are typically complex three-dimensional structures that the optics cannot yet replicate. On the other hand, the underlying physics is similar. Elementary building blocks consisting of atoms and tightly bound molecules self-assemble to form supramolecules through interactions mediated by weak, long-range forces. The mode-locked fibre laser reported here, a fast one-dimensional optical platform with a single degree of freedom^13^, may be useful for emulating complex process in many-body biochemical and biological systems.

Within the taxonomy of nonlinear optics, soliton supramolecules are structurally protected solutions of dissipative nonlinear optical systems^22,47^. These systems include mode-locked fibre lasers^13,48^, optical bit-storage fibre loops^4–6^ or optical fibre telecommunication systems with non-trivial Kerr nonlinearities^1,2,49^. By periodically introducing control elements^50^ into supramolecular soliton systems with non-instantaneous nonlinearities, it may be possible to manipulate the supramolecules and form flexible, highly-ordered structures that are immune to perturbations. The soliton supramolecules reported here can be constructed from many different multi-soliton bound states, making it possible to encode information in non-binary formats using both long-range and short-range interactions. The ability to tailor long-range interactions between solitons in an optomechanical lattice may permit synthesis and control of highly-ordered, macroscopic optical structures, providing a promising platform for studying complex soliton molecules (e.g. their formation^22,51^, dissociation^13,52^, and vibrational modes^45,53^) and optically simulating many-body systems with particle-like properties. The ability to synthesize highly ordered multi-soliton sequences may also be useful for improving laser micromachining^54^. The enriched dynamics of controllable long-range soliton interactions, and their analogies with the behaviour of chemical supramolecules (e.g. self-healing and self-replication), are also interesting research topics. Introducing spatiotemporal nonlinearities using multimode fibres^48^ may in the future permit the formation of three-dimensional optical supramolecules, further enriching the physics and applications of the current system.

## Methods

### Mode-locked fibre laser

The experimental platform is an optoacoustically mode-locked soliton fibre laser, with a pulse repetition rate that is locked to an acoustic resonance in the core of a photonic crystal fibre (PCF) inserted in the laser cavity. The cavity has net anomalous dispersion, ensuring that the fibre laser operates in the soliton regime. To achieve harmonic mode-locking and generate soliton supramolecules in the laser cavity, it is important to set a proper working point. First, the delay line in the cavity is adjusted so that a specific harmonic order of the cavity FSR falls within the optoacoustic gain spectrum of the PCF. Second, all the three fibre polarization controllers (FPCs) in the laser cavity are adjusted so that nonlinear polarization rotation (NPR) can induce an intensity-dependent cavity loss. Finally, the laser pump power and the intra-cavity attenuator are adjusted to enable self-assembly of a supramolecular structure. Once the desired soliton supramolecule is obtained, no further adjustment or any other stabilization technique is needed for long-term preservation of the supramolecular soliton pattern.

### Diagnostic set-up

A time-domain trace of the laser output is acquired using a 30-GHz photodetector and a 33-GHz oscilloscope (OSC). The response time is ~20 ps, limiting the minimum resolvable temporal features in all the plots recorded using the OSC. The timing jitter of the OSC in sampling is ~2 ps, which sets the measurement error in obtaining the fine structure of the supramolecule. The duration of individual solitons is measured using a second-harmonic autocorrelator with a time resolution of 20 fs. The optical spectrum at the laser output is measured using an optical spectrum analyser with a resolution of 0.01 nm. We also performed measurements using time-stretched dispersive Fourier transformation (TS-DFT), using several-km-long SMF-28 fibre to characterize the soliton molecules in the supramolecules.

### Tailoring of long-range interactions

The long-range interactions involved in soliton supramolecules can be tailored by tuning either the intensity of dispersive wave and thus the repulsive force between solitons, or the amplitude of the acoustic vibration in the PCF core, corresponding to the force of attraction between solitons. In order to tune the dispersive-wave intensity as shown in Fig. 4, we adjusted the intra-cavity tunable attenuator (TA), leading to higher cavity gain and therefore a stronger gain filtering effect, which significantly suppressed the Kelly-sideband intensity. During this dispersive-wave tuning process, the soliton spectral bandwidth remained almost unchanged due to strong gain saturation in the EDFA. See Supplementary Note 7 for more effects of dispersive-wave tailoring.

### Adding and removing solitons

Addition and removal of individual solitons is achieved by strongly perturbing the laser pump power. In the experiments, the output power of the pump laser diode is controlled using an electric pulse generator. The modulation responsivity of the pump laser diode is 106 mW/V, so that a 1 V variation in the driving electrical signal leads to a 106 mW variation in the laser output power. Before adding or removing solitons, it is important first to adjust the working point of the laser so that the supramolecular structure is stable.

## Supplementary information

Supplementary Information

## Data Availability

The data that support the plots within this paper and other findings of this study are available from the corresponding authors upon reasonable request.

## Code availability

The code we used in this paper is available from the corresponding author upon reasonable request.

## References

[1] Haus HA, Wong WS (1996). Solitons in optical communications. Rev. Mod. Phys..

[2] Nakazawa M, Yamada E, Kubota H, Suzuki K (1991). 10 Gbit/s soliton data transmission over one million kilometres. Electron. Lett..

[3] Barland S (2002). Cavity solitons as pixels in semiconductor microcavities. Nature.

[4] Leo F (2010). Temporal cavity solitons in one-dimensional Kerr media as bits in an all-optical buffer. Nat. Photon.

[5] Pang M, He W, Jiang X, Russell PStJ (2016). All-optical bit storage in a fibre laser by optomechanically bound states of solitons. Nat. Photonics.

[6] Mecozzi A, Kath WL, Kumar P, Goedde CG (1994). Long-term storage of a soliton bit stream by use of phase-sensitive amplification. Opt. Lett..

[7] Firth WJ, Weiss CO (2002). Cavity and feedback solitons. Opt. Photonics N..

[8] Tanguy Y, Ackemann T, Firth WJ, Jäger R (2008). Realization of a semiconductor-based cavity soliton laser. Phys. Rev. Lett..

[9] Garbin B, Javaloyes J, Tissoni G, Barland S (2015). Topological solitons as addressable phase bits in a driven laser. Nat. Commun..

[10] Jang JK, Erkintalo M, Coen S, Murdoch SG (2015). Temporal tweezing of light through the trapping and manipulation of temporal cavity solitons. Nat. Commun..

[11] Jang JK, Erkintalo M, Murdoch SG, Coen S (2015). Writing and erasing of temporal cavity solitons by direct phase modulation of the cavity driving field. Opt. Lett..

[12] Herr T (2014). Temporal solitons in optical microresonators. Nat. Photon.

[13] Grelu P, Akhmediev N (2012). Dissipative solitons for mode-locked lasers. Nat. Photon.

[14] Marconi M (2015). Control and generation of localized pulses in passively mode-locked semiconductor lasers. IEEE J. Sel. Top. Quantum Electron..

[15] Malomed BA (1991). Bound solitons in the nonlinear Schrodinger-Ginzburg-Landau equation. Phys. Rev. A.

[16] Akhmediev NN, Ankiewicz A, Soto-Crespo JM (1997). Multisoliton solutions of the complex Ginzburg-Landau equation. Phys. Rev. Lett..

[17] Stratmann M, Pagel T, Mitschke F (2005). Experimental observation of temporal soliton molecules. Phys. Rev. Lett..

[18] Cole DC, Lamb ES, Del’Haye P, Diddams SA, Papp SB (2017). Soliton crystals in Kerr resonators. Nat. Photon.

[19] Jang JK, Erkintalo M, Murdoch SG, Coen S (2013). Ultraweak long-range interactions of solitons observed over astronomical distances. Nat. Photon.

[20] Wang Y (2017). Universal mechanism for the binding of temporal cavity solitons. Optica.

[21] Weill R, Bekker A, Smulakovsky V, Fischer B, Gat O (2016). Noise-mediated Casimir-like pulse interaction mechanism in lasers. Optica.

[22] Herink G, Kurtz F, Jalali B, Solli DR, Ropers C (2017). Real-time spectral interferometry probes the internal dynamics of femtosecond soliton molecules. Science.

[23] Wang ZQ, Nithyanandan K, Coillet A, Tchofo-Dinda P, Grelu P (2019). Optical soliton molecular complexes in a passively mode-locked fibre laser. Nat. Commun..

[24] Amrani F (2011). Passive harmonic mode locking of soliton crystals. Opt. Lett..

[25] Sulimany K (2018). Bidirectional soliton rain dynamics induced by Casimir-like interactions in a graphene mode-locked fiber laser. Phys. Rev. Lett..

[26] Socci L, Romagnoli M (1999). Long-range soliton interactions in periodically amplified fiber links. J. Opt. Soc. Am. B.

[27] Loh WH, Afanasjev VV, Payne DN, Grudinin AB (1994). Soliton interaction in the presence of a weak nonsoliton component. Opt. Lett..

[28] Grudinin AB, Gray S (1997). Passive harmonic mode locking in soliton fiber lasers. J. Opt. Soc. Am. B.

[29] Rotschild C, Alfassi B, Cohen O, Segev M (2006). Long-range interactions between optical solitons. Nat. Phys..

[30] Amrani F (2009). Passively mode-locked erbium-doped double-clad fiber laser operating at the 322nd harmonic. Opt. Lett..

[31] Kutz JN, Collings BC, Bergman K, Knox WH (1998). Stabilized pulse spacing in soliton lasers due to gain depletion and recovery. IEEE J. Quantum Electron..

[32] Pang M (2015). Stable subpicosecond soliton fiber laser passively mode-locked by gigahertz acoustic resonance in photonic crystal fiber core. Optica.

[33] He W, Pang M, Russell PStJ (2015). Wideband-tunable soliton fiber laser mode-locked at 1.88 GHz by optoacoustic interactions in solid-core PCF. Opt. Express.

[34] He W, Pang M, Menyuk CR, Russell PStJ (2016). Sub-100-fs 1.87 GHz mode-locked fiber laser using stretched-soliton effects. Optica.

[35] McLaughlin CK, Hamblin GD, Sleiman HF (2011). Supramolecular DNA assembly. Chem. Soc. Rev..

[36] Service RF (2002). Strength in numbers. Science.

[37] Kang MS, Nazarkin A, Brenn A, Russell PStJ (2009). Tightly trapped acoustic phonons in photonic crystal fibres as highly nonlinear artificial Raman oscillators. Nat. Phys..

[38] Pilipetskii AN, Golovchenko EA, Menyuk CR (1995). Acoustic effect in passively mode-locked fiber ring lasers. Opt. Lett..

[39] Noske DU, Pandit N, Taylor JR (1992). Source of spectral and temporal instability in soliton fiber lasers. Opt. Lett..

[40] Kelly SMJ (1992). Characteristic sideband instability of periodically amplified average soliton. Electron. Lett..

[41] Bondeson A, Lisak M, Anderson D (1979). Soliton perturbations: a variational principle for the soliton parameters. Phys. Scr..

[42] Kodama Y, Hasegawa A (1992). Generation of asymptotically stable optical solitons and suppression of the Gordon–Haus effect. Opt. Lett..

[43] Gordon JP, Haus HA (1986). Random walk of coherently amplified solitons in optical fiber transmission. Opt. Lett..

[44] Tamura K, Ippen EP, Haus HA, Nelson LE (1993). 77-fs pulse generation from a stretched-pulse mode-locked all-fiberring laser. Opt. Lett..

[45] Krupa K, Nithyanandan K, Andral U, Tchofo-Dinda P, Grelu P (2017). Real-time observation of internal motion within ultrafast dissipative optical soliton molecules. Phys. Rev. Lett..

[46] Herink G, Jalali B, Ropers C, Solli DR (2016). Resolving the build-up of femtosecond mode-locking with single-shot spectroscopy at 90-MHz frame rate. Nat. Photon.

[47] Carmon T (2001). Rotating propeller solitons. Phys. Rev. Lett..

[48] Wright LG, Christodoulides DN, Wise FW (2017). Spatiotemporal mode-locking in multimode fiber lasers. Science.

[49] Mitra PP, Stark JB (2001). Nonlinear limits to the information capacity of optical fibre communications. Nature.

[50] Falkovich G, Kolokolov I, Lebedev V, Mezentsev V, Turitsyn S (2004). Non-Gaussian error probability in optical soliton transmission. Phys. D. Nonlinear Phenom..

[51] Soto-Crespo JM, Grelu P, Akhmediev N, Devine N (2007). Soliton complexes in dissipative systems: vibrating, shaking, and mixed soliton pairs. Phys. Rev. E.

[52] Wang X (2019). Real-time observation of dissociation dynamics within a pulsating soliton molecule. Opt. Express.

[53] Shi H, Song Y, Wang C, Zhao L, Hu M (2018). Observation of subfemtosecond fluctuations of the pulse separation in a soliton molecule. Opt. Lett..

[54] Kerse C (2016). Ablation-cooled material removal with ultrafast bursts of pulses. Nature.

