# Supplementary material for: Formation of optical supramolecular structures in a fibre laser by tailoring long-range soliton interactions
Source: Nat Commun. 2019 Dec 17;10:5756. doi: 10.1038/s41467-019-13746-6 (PMC6917763; doi:10.1038/s41467-019-13746-6)
Supplement: Supplementary file 1 — Supplementary Information [file 41467_2019_13746_MOESM1_ESM.pdf]

Supplementary Information for

“Formation of optical supramolecular structures in a fibre laser by tailoring long-range soliton interactions”

W. He et al.

Supplementary Content

Supplementary Note 1: Experimental set-up --- 2

Supplementary Note 2: Theory of long-range forces between optical solitons ----------------- 3

Supplementary Note 3: Theoretical modelling on pulse timing jitter ---------------------------- 7

Supplementary Note 4: Long-term preservation of soliton supramolecules -------------------- 10

Supplementary Note 5: Uncorrelated phase relations --- 11

Supplementary Note 6: Uniform soliton supramolecules -- 12

Supplementary Note 7: Directions of the repulsive forces -- 17

Supplementary Note 8: Details of elementary diversity -- 19

Supplementary Note 9: Tuning of inter-soliton spacing --- 30

Supplementary Note 10: Self-assembling dynamics --- 32

Supplementary References --- 37

Supplementary Note 1: Experimental set-up

In Supplementary Figure 1, we sketch the experimental set-up, including a fibre laser cavity (right) and a diagnostic set-up (left). In the optoacoustically mode-locked soliton fibre laser[1–4], the 1.2-m-long erbium-doped gain fibre is pumped by two laser diodes at 980 nm with a combined pump power of 1.5 W. The 2-m-long solid-core silica photonic crystal fibre (PCF) that is spliced into the laser cavity has a core diameter of 1.95 µm, with the R_{01} mechanical core resonance at 1.887 GHz[5] and an optical birefringence of 1.5×10^{-4}. The total insertion loss of the PCF section is 1.9 dB. An optical isolator (ISO) ensures the unidirectional operation of the laser. Three fibre polarization controllers (FPCs) are used inside the laser cavity, among which FPC-1 and -3 are used for enabling the self-start of the laser mode-locking through nonlinear polarization rotation (NPR)[6,7], while the FPC-2 is adjusted to launch the linearly polarized laser light into one principal axis of the PCF[1]. A tunable attenuator (TA) is inserted in the cavity to adjust the cavity loss, which can be tuned from ~ 6 dB to ~ 20 dB with a resolution of 0.02 dB. A tunable delay line (TD) is used to adjust the cavity length, with a tuning range of 0.15 m and a resolution of 3 µm. The total cavity length is ~17 m, corresponding to a free spectral range (FSR) of ~12.2 MHz. The cavity average dispersion is about –0.046 ps^2/m, ensuring operation of this laser in the soliton regime[8,9]. When the laser is optoacoustically mode-locked at the PCF core resonance (~1.88 GHz in practice), the laser cavity is effectively divided into 154 time-slots by the optically-driven GHz-rate mechanical vibration in the PCF core, providing an effective optomechanical lattice[4]. Within each time-slot (mechanical vibration cycle) of the lattice, one long-range multi-soliton unit can be trapped, and hundreds to thousands of laser solitons can be incorporated in the supramolecular assembly. In the diagnostic set-up, the 10% laser output from the 90:10 coupler is divided into 4 parts using three couplers, which are connected to a fast oscilloscope (OSC), a second-harmonic autocorrelator (AC), an optical spectrum analyzer and a time-stretched dispersive Fourier transform (TS-DFT) set-up. In the TS-DFT set-up, a few-km single-mode fibre (SMF) is employed to stretch the laser pulses in time domain and a low-noise fast photodetector to detect the stretched signal. A 90-cm-long dispersion-compensation fibre (DCF) is used before the AC for pulse chirp compensation in order to measure the pulse duration.

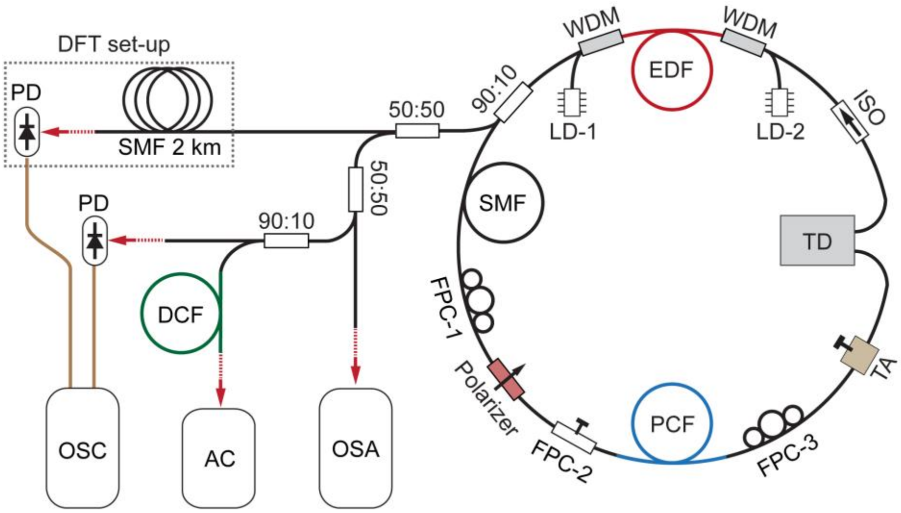

Supplementary Figure 1 | Experimental set-up. (Right Part) The unidirectional soliton fibre laser and (Left part) the diagnostic set-up. The laser output is recorded using a fast oscilloscope (OSC) and a high-resolution optical spectrum analyzer (OSA). An autocorrelator (AC) is used to measure the pulse duration. EDF: Erbium-doped fibre; WDM: wavelength division multiplexer; LD: laser diode; SMF: single-mode fibre; PC: polarization controller; PCF: photonic crystal fibre; TA: tunable attenuator; TD: tunable delay line; ISO: optical isolator; PD: photo detector; DCF: dispersion-compensation fibre.

Supplementary Note 2: Theory of long-range forces between optical solitons

We present here the theoretical model for the long-range, inter-soliton interactions which lead to long-range binding of multiple solitons within each time-slot of the optomechanical lattice. We consider the simplest all-double-soliton (ADS) supramolecule composed of "uniform" double-soliton units in all the time-slots of the lattice (see Fig. 2c and Fig. 4 in the main text, Supplementary Figure 8 in Supplementary Note 6, and Supplementary Figure 21 in Supplementary Note 9). Supplementary Figure 2 sketches one time-slot (acoustic vibration cycle) of the ADS supramolecule that accommodates two solitons. In a frame moving with the reference soliton (e.g. the first soliton in Supplementary Figure 2), the first soliton is located at t_1 and the second soliton at t_2, with the spacing $\Delta t = t_1 - t_2$. The effective force of attraction between these two solitons results in a slower group velocity of the second soliton than the first, which leads to a delay of the second soliton relative to the first one during propagation. Conversely, a faster velocity of the second soliton would result from an effective force of repulsion. When the soliton spacing is hundreds of times longer than the duration of individual solitons, soliton interactions become long-range, distinct from direct soliton interactions in conventional soliton molecules[10–13] due to the overlap of pulse tails. The stable separation between two solitons in one time-slot of the optomechanical lattice is due to the balance of two long-range forces.

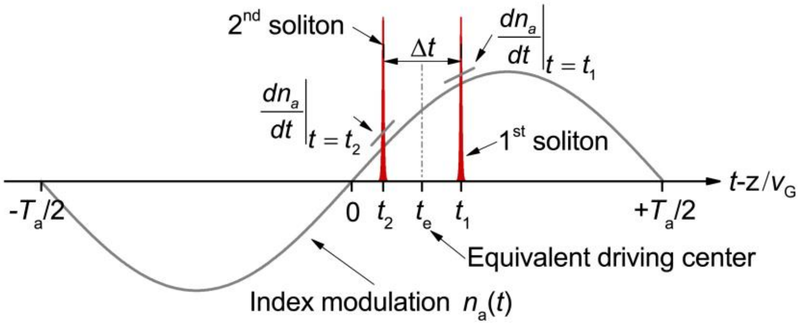

Supplementary Figure 2 | One double-soliton unit of the ADS supramolecule and the optically-driven index modulation within one time-slot of the optomechanical lattice. In one time-slot, two solitons locate at t_1 and t_2, giving a soliton spacing of Δt and an equivalent driving center of t_e. When propagating in the PCF core, the two solitons experience different refractive index slopes.

Force of attraction due to the optomechanical effect

When the ADS supramolecule is circulating in the laser cavity with a lattice frequency of Ω_a, it coherently drives the mechanical core resonance in the PCF. The optically-driven mechanical vibration, in the form of a refractive index modulation, acts back on the driving solitons by varying their carrier frequencies[4]. In practice, we only consider the LP_{01} optical mode and the R_{01} mechanical resonance in the PCF[4,5]. The R_{01} mechanical mode in the PCF has a resonant frequency of Ω_{01} and a mechanical bandwidth of Γ_B. In a reference frame moving with the soliton supramolecule, the mechanical vibration in the PCF core driven by the ADS supramolecule can be expressed as[4]:

$$b(t) = \frac{\gamma_e |Q| P_{av} \cos(\Omega_a \Delta t / 2)}{2\pi c n_0 A_{eff} \Omega_a \sqrt{4\delta_\Omega^2 + \Gamma_B^2}} \sin(\Omega_a t), \tag{1}$$

where $b(t)$ is the material density variation as a function of time, γ_e is the electrostrictive constant of silica, c is the speed of light in vacuum, n_0 is the refractive index of silica, A_{eff} is the effective mode area of the LP_{01} optical mode, δ_Ω is the frequency off-set ($\delta_\Omega = \Omega_a - \Omega_{01}$), and P_{av} is the average optical power

in the PCF. The overlap integral Q is defined as $Q = \left\langle \rho_{01} \nabla_{\perp}^{2} \left| E_{01} \right|^{2} \right\rangle / \left\langle \rho_{01}^{2} \right\rangle$, where ρ_{01} and E_{01} are the transverse field distributions of the R_{01} mechanical mode and the LP_{01} optical mode. The equivalent driving center (t_e) of the double-soliton unit relative to the excited mechanical vibration can be expressed as[4]

$$t_{\mathrm{e}} = \frac{1}{\Omega_{\mathrm{a}}} \tan^{-1}\left(-\frac{2\delta_{\Omega}}{\Gamma_{\mathrm{B}}} \right), \tag{2}$$

The timing of the first and the second solitons in this frame can then be expressed as $t_1 = t_e + \Delta t/2$ and $t_2 = t_e - \Delta t/2$. The optically-driven mechanical vibration modulates the effective refractive index of the LP_{01} optical mode through the stress-optical effect, which can be expressed as

$$n_{\mathrm{a}}(t) = \frac{\gamma_{\mathrm{e}}\Theta}{2n_0\rho_0} b(t) = C_{\mathrm{a}} \frac{P_{\mathrm{av}}}{\Omega_{\mathrm{a}}\sqrt{4\delta_{\Omega}^{2} + \Gamma_{\mathrm{B}}^{2}}} \cos\left(\frac{\Omega_{\mathrm{a}}\Delta t}{2} \right) \sin\left(\Omega_{\mathrm{a}} t \right), \tag{3}$$

where $C_{\mathrm{a}} = \gamma_{\mathrm{e}}^{2} \left| Q \right| \Theta \big/ \left(4\pi c n_0^{2} A_{\mathrm{eff}} \rho_0 \right)$ and ρ_0 is the material density of silica. The overlap integral[5] (Θ) between the normalized intensity field of the LP_{01} optical mode and the normalized density field of the R_{01} mechanical mode can be defined as $\Theta = \left\langle \rho_{01} \left| E_{01} \right|^{2} \right\rangle / \left\langle \rho_{01}^{2} \right\rangle$.

Back-action of the mechanical vibration results in a carrier frequency shift of the driving soliton, which is directly related to the refractive index slope that the soliton experiences[14], expressed as

$$\frac{\partial \omega_{\mathrm{s}}}{\partial z} = -\frac{\omega_{\mathrm{s}}}{c} \frac{\partial n_{\mathrm{a}}}{\partial t}, \tag{4}$$

where ω_s is the soliton carrier frequency. As shown in Supplementary Figure 2, in one mechanical cycle, the two solitons experience different index slopes, leading to a carrier frequency difference between the two solitons. Using Supplementary Equation 3 and 4, this frequency difference after a single-trip propagation in the PCF can be expressed as:

$$\Delta\omega_{\mathrm{a}} = \delta\omega_{\mathrm{s2}} - \delta\omega_{\mathrm{s1}} = \frac{2C_{\mathrm{a}}\omega_{\mathrm{s}}P_{\mathrm{av}}L_{\mathrm{PCF}}}{c} \frac{\delta_{\Omega}}{4\delta_{\Omega}^{2} + \Gamma_{\mathrm{B}}^{2}} \sin\left(\Omega_{\mathrm{a}}\Delta t \right), \tag{5}$$

where $\delta\omega_{s1}$ and $\delta\omega_{s2}$ are the carrier frequency shifts of the first and second solitons due to the optomechanical effect in the PCF, and L_{PCF} is the PCF length. When the lattice frequency of the soliton supramolecule is lower than the mechanical resonance frequency in the PCF ($\delta_\Omega < 0$), $\Delta\omega_a$ remains negative, which means that the optomechanical effect leads to a lower carrier frequency of the second soliton than that of the first one. In a fibre cavity with anomalous average dispersion, solitons with lower frequency propagate at a lower group velocity, which delays the second soliton relative to the first one during propagation, leading to an effective force of attraction between the two solitons.

Force of repulsion due to dispersive wave perturbations

A competing long-range interaction between the two solitons results from dispersive waves that are shed from the solitons. These dispersive waves can extend in the time domain to the neighboring solitons and perturb them through cross-phase modulation[15,16]. Dispersive waves, corresponding to Kelly sidebands on the soliton laser spectrum, appear at discrete frequencies (see Supplementary Figure 3a). In the systems for long-distance soliton telecommunications and soliton fibre lasers, the existence of dispersive waves is a generic phenomenon that is due to periodic disturbances arising from the discrete dispersion, nonlinearity, gain and loss in these systems[17–19]. During propagation, optical solitons coherently transfer their energy to several discrete frequency components (sidebands). Due to the gain filtering that occurs

in the EDFA[20], those sidebands would always experience a net loss in the cavity. During one cavity round trip, such net cavity loss must be balanced by a nonlinear gain, which is due to the energy conversion from the solitons to these sidebands. This balance between gain and loss determines the steady-state intensities of these sidebands. In practice, due to the uneven gain profile in the EDFA[20] and higher-order dispersion in the fibre cavity, Kelly sidebands at different orders have usually different intensities[19]. The situation we consider in the main text is that the m=–1 order sideband has the dominant intensity as shown in Supplementary Figure 3a, while other possibilities are demonstrated in Supplementary Note 7 below.

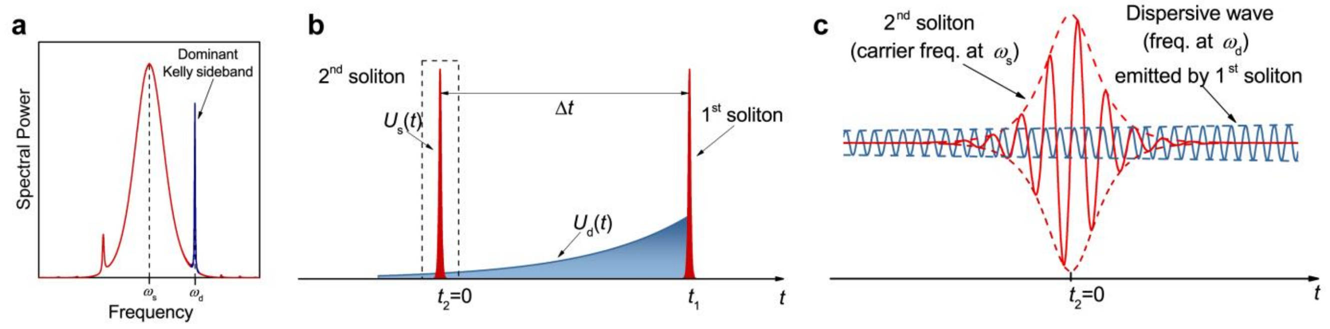

Supplementary Figure 3 | Long-range soliton interactions due to dispersive wave perturbations. **a**, A typical soliton spectrum with a dominant (m=–1 order) Kelly sideband at ω_d (ω_d>ω_s). **b**, This dominant sideband corresponds, in the time domain, to a packet of dispersive wave. The dispersive wave shed from the first soliton has a faster group velocity than the soliton, and extends to the second soliton in the time domain. **c**, Interactions between the second soliton and the dispersive wave shed from the first soliton shift the carrier frequency of the second soliton.

This dominant sideband corresponds, in the time domain, to a packet of dispersive wave shed from solitons in the laser cavity. Within one double-soliton unit, the dispersive wave shed from the first soliton (see Supplementary Figure 3b) propagates faster than the solitons due to its higher carrier frequency, and its exponentially-decaying envelope is due to the net cavity-loss that it experiences in the cavity. In this moving frame, the second soliton can be expressed as:

$$U_s(t) = A_s \operatorname{sech}[t/\tau_s]\exp[i\omega_s t - i\varphi_s(z)] = A_s u_s(t), \tag{6}$$

where A_s is the soliton peak amplitude, $u_s(t)$ the normalized profile, τ_s the pulse width, and φ_s the phase of the second soliton varying with propagation length. For simplicity, we neglect the variation of the soliton envelope within one round trip, which means that both the soliton duration (thus the bandwidth) and the peak soliton amplitude are constant during propagation inside the laser cavity.

In the reference frame moving with the second soliton ($t_2 = 0$), the dispersive wave shed from the first soliton can be expressed as:

$$U_d(t) = A_d \exp[h(t-\Delta t)]\exp[i\omega_d(t-\Delta t) - i\varphi_d(z)] = A_s u_d, \tag{7}$$

where A_d is the dispersive wave amplitude, h the decay rate, ω_d the carrier frequency, and φ_d the phase of the dispersive wave. Then u_d is the dispersive wave waveform normalized to the soliton peak amplitude. The phase $\varphi_d(z)$ includes the propagation term of the dispersive wave, and varies as the wave propagates in the cavity. We estimated A_d and ω_d using the peak intensity and central frequency of the dominant sideband, and we calculated h using the spectral bandwidth of the sideband.

According to soliton perturbation theory[15,21], the carrier frequency of the second soliton varies due to the dispersive wave shed from the first soliton (see Supplementary Figure 3c) according to the expression

$$\frac{d\omega_{\mathrm{s}}}{dz} = \frac{1}{\tau_{\mathrm{s}} L_{\mathrm{D}}} \mathrm{Im}\left\{\int_{-\infty}^{+\infty} u_{\mathrm{s}}^{*}(t)\tanh(t/\tau_{\mathrm{s}}) u_{\mathrm{d}}(t) dt\right\}, \tag{8}$$

where L_{D} is the dispersion length of the soliton. After substituting Supplementary Equation 6 and 7 into Supplementary Equation 8, we obtain

$$\frac{d\omega_{s}}{dz} = \frac{A_{d}}{A_{\mathrm{s}}\tau_{\mathrm{s}} L_{\mathrm{D}}} B(\Delta\omega)\exp(-h\Delta t)\cos\left[\Delta\varphi(z)\right], \tag{9a}$$

$$\begin{aligned} B(\Delta\omega) &= \int_{-\infty}^{+\infty} \mathrm{sech}(t/\tau_{\mathrm{s}})\tanh(t/\tau_{\mathrm{s}})\sin(\Delta\omega t)dt \\ &= \pi\Delta\omega\tau_{\mathrm{s}}\,\mathrm{sech}(\pi\Delta\omega\tau_{\mathrm{s}}/2), \end{aligned} \tag{9b}$$

where $\Delta\omega = \omega_{\mathrm{d}} - \omega_{\mathrm{s}}$ is the carrier frequency difference, and $\Delta\varphi(z) = \varphi_{\mathrm{s}}(z) - \varphi_{\mathrm{d}}(z)$ is the phase difference between the soliton and the dispersive wave. In practice, the integral $B(\Delta\omega)$ is a constant close to 1, and the accumulated frequency shift of the second soliton after each cavity round trip (RT) can be expressed as

$$\Delta\omega_{\mathrm{d}}^{\mathrm{RT}} = \frac{A_{\mathrm{d}}}{A_{\mathrm{s}}\tau_{\mathrm{s}} L_{\mathrm{D}}} B(\Delta\omega)\exp(-h\Delta t)\int_{z_0}^{z_0+L_{\mathrm{c}}} \cos\left[\Delta\varphi(z)\right]dz, \tag{10}$$

where z_0 is the starting point for integral in the cavity, and L_{c} is the cavity length. In Supplementary Equation 10, we assume an invariant intensity of the dispersive wave during its propagation in the cavity. Otherwise, we would have to include an intensity distribution $A_{\mathrm{d}}(z)$ within this integral.

The phase-matching condition for dispersive wave generations in mode-locked fibre lasers[17–19] requires that the accumulated phase difference between solitons and dispersive waves over one cavity round trip should be integer multiples of 2π. For the $m = -1$ order dispersive wave, this accumulated phase difference should be -2π. If we define the phase difference at an arbitrary position (z_0) in the cavity as $\Delta\varphi_0$, we obtain

$$\Delta\varphi(z) = \int_{z_0}^{z}\left[\frac{1}{2}\beta_2(z)\Delta\omega^2 - \frac{1}{2}\gamma(z)\left|A_{\mathrm{s}}(z)\right|^2\right]dz + \Delta\varphi_0, \tag{11a}$$

$$\Delta\varphi(z + L_{\mathrm{c}}) - \Delta\varphi(z) = -2\pi, \tag{11b}$$

where $\beta_2(z)$ and $\gamma(z)$ are the dispersion and nonlinearity in the laser cavity. Substituting Supplementary Equation 11a into Supplementary Equation 10, we obtain:

$$\Delta\omega_{\mathrm{d}}^{\mathrm{RT}} = \frac{A_{\mathrm{d}}}{A_{\mathrm{s}}\tau_{\mathrm{s}} L_{\mathrm{D}}} B(\Delta\omega)\exp(-h\Delta t)\psi(\Delta\varphi_0), \tag{12a}$$

$$\psi(\Delta\varphi_0) = \int_{z_0}^{z_0+L_{\mathrm{c}}} \cos\left\{\int_{z_0}^{z}\left[\frac{1}{2}\beta_2(z)\Delta\omega^2 - \frac{1}{2}\gamma(z)\left|A_{\mathrm{s}}(z)\right|^2\right]dz + \Delta\varphi_0\right\}dz. \tag{12b}$$

Using Supplementary Equation 12b, it can be seen that when both the dispersion and nonlinearity in the cavity are fixed functions of z, the integral ψ only depends on the initial phase difference ($\Delta\varphi_0$), which determines the sign of the carrier frequency shift of the perturbed soliton. In order to form stable double-soliton units, an effective force of repulsion due to this dispersive wave perturbation is necessary to balance the force of attraction due to the optomechanical effect. According to Supplementary Equation 12, this balance requires a special phase relationship between the dispersive wave shed from the first soliton and the second soliton to ensure a positive $\Delta\omega_{\mathrm{d}}^{\mathrm{RT}}$. Acting in concert with the anomalous average

dispersion of the laser cavity, this positive $\Delta\omega_{\mathrm{d}}^{\mathrm{RT}}$ would lead to a faster group velocity of the second soliton than that of the first one, resulting in an effective force of repulsion between the two solitons. The existence of this phase relationship has been verified in the experiments. We observe in the experiments clear interferometric fringes on these optical spectra, which appeared only in the vicinity of the dominant sideband, indicating the phase locking between the dispersive waves and the perturbed solitons, as shown in Fig. 4b in the main text, as well as Supplementary Figure 8h, 9h, 12c, and, 21a in the following Supplementary Notes.

We also observe in the experiments that the interferometric fringes (and thus corresponding phase locking) are very robust over time. The stability of this phase locking mechanism and its robustness in the presence of noises have not been rigorously investigated. At present, we use this phase relation as a basic assumption of the theoretical derivation, and we leave a full explanation of its origin as an open question.

Balance of the two long-range forces

The build-up of the long-range binding of the two solitons in one time-slot of the optomechanical lattice is based on the precise balance between the effective long-range forces of attraction and repulsion. Over each cavity round trip, the overall carrier frequency shift of the second soliton relative to the first should equal to zero (i.e. $\Delta\omega_{\mathrm{a}}+\Delta\omega_{\mathrm{d}}^{\mathrm{RT}}=0$). Therefore the two solitons always have the same averaged group velocity during propagation and travel together with invariant spacing between them. Using Supplementary Equation 5 and 12a, this balance can be expressed as:

$$-\frac{2C_{\mathrm{a}}\omega_{\mathrm{s}}P_{\mathrm{av}}L_{\mathrm{PCF}}}{c}\frac{\delta_{\Omega}}{4\delta_{\Omega}^{2}+\Gamma_{\mathrm{B}}^{2}}\sin\left(\Omega_{\mathrm{a}}\Delta t\right)=\frac{A_{\mathrm{d}}}{A_{\mathrm{s}}\tau_{\mathrm{s}}L_{\mathrm{D}}}A_{\mathrm{d}}B\left(\Delta\omega\right)\exp\left(-h\Delta t\right)\psi(\Delta\varphi_{0})\,. \tag{13}$$

While the right side of the equation has a positive sign corresponding to a force of repulsion, a negative detuning of the lattice frequency from the mechanical resonance frequency ($\delta_{\Omega}<0$) ensures that the optomechanical effect in the PCF creates an effective force of attraction between the two solitons. The theoretical fitting curves (Fig. 4c in the main text and Supplementary Figure 21c in Supplementary Note 9) are based on Supplementary Equation 13.

When the system parameters are fixed, the intensity of the force of attraction on the left side of Supplementary Equation 13 increases as the soliton spacing (Δt) increases, while the intensity of the force of repulsion on the right side of Supplementary Equation 13 decreases as the soliton spacing increases. Such line shapes are necessary to form a temporal trapping potential for the second soliton (see Fig. 1d and 1e in the main text), which makes the long-range binding of solitons robust. The existence of this trapping potential is revealed during the dynamic process of adding or removing individual solitons to the supramolecular structure, in which we observe an adjustment of the soliton position within the time-slot towards the balanced position (see Supplementary Note 10 below).

Supplementary Note 3: Theoretical modelling on pulse timing jitter

As demonstrated in Fig. 2b in the main text, the pulse timing jitter in the soliton supramolecule did not grow with time, which is due to the presence of many balanced positions in the supramolecular structure within which optical solitons can settle. This phenomenon is quite different from that in the conventional soliton fiber laser or soliton telecommunications system in which the timing jitter typically accumulates during long-distance propagation in the optical fibre. We establish here a simple model that describes the timing jitter of the soliton spacing in the presence of different types of "forces", including the long-range inter-soliton force, the damping-related force caused by the gain filtering effect, and the noise-related

force coming from the spontaneous emission of the EDFA ("heat bath"). In order to simplify the mathematical expression of the following model, we will switch from the time-domain description to the spatial-domain description, while still using the moving frame, i.e. the spatial coordinate z is related to the time coordinate t via $z = z' - v_g t$, in which z' is the actual spatial coordinate along the fibre. Then, the balanced pulse spacing (t_0) in the time domain (see Fig. 1e in the main text) is directly related to the spatial pulse spacing of z_0 (see Supplementary Figure 4a) through $z_0 = -v_g t_0$.

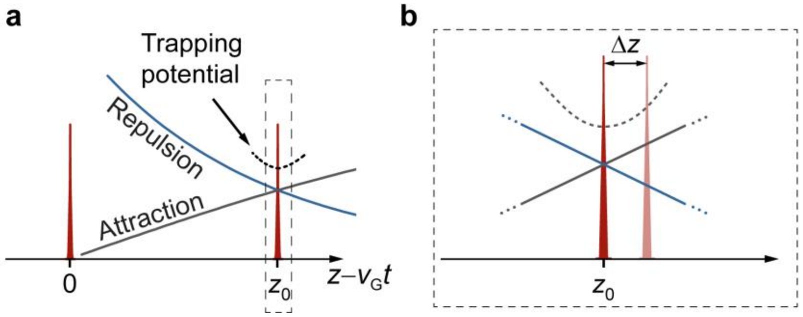

Supplementary Figure 4 | a, The trapping potential of the second soliton is formed at the balance position (z_0) of two long-range forces. **b**, The slight deviation from the equilibrium position (Δz) would lead to a net restoring force.

The perturbations of the soliton carrier frequency due to optoacoustic effects and dispersive waves described in Supplementary Note 2 can then be linearized in the close vicinity of the balanced position z_0 (see Supplementary Figure 4b) to yield

$$\left(\frac{d\Delta\omega}{dt}\right)_{\mathrm{A}} = -K_{\mathrm{A}} z , \tag{14a}$$

$$\left(\frac{d\Delta\omega}{dt}\right)_{\mathrm{D}} = C_0 - K_{\mathrm{D}} z , \tag{14b}$$

The two opposite frequency shifts should precisely cancel each other at the equilibrium position $z_0 = C_0/(K_{\mathrm{A}} + K_{\mathrm{D}})$. If the soliton has a slight spatial deviation $\Delta z = z - z_0$ from the equilibrium position, the soliton would have a net frequency shift during propagation, so that

$$\frac{d\Delta\omega}{dt} = -K\Delta z , \tag{15}$$

where $K = K_{\mathrm{A}} + K_{\mathrm{D}}$. The spatial deviation Δz is related to the group velocity difference Δv_{g} between the two solitons through

$$\frac{d\Delta z}{dt} = \Delta v_{\mathrm{g}} , \tag{16}$$

and Δv_{g} is related to $\Delta\omega$ through the cavity group velocity dispersion, i.e.

$$\Delta v_{\mathrm{g}} = B\Delta\omega , \text{ where } B = -\beta_2 v_{\mathrm{g}}^2 , \tag{17}$$

After we substitute Supplementary Equation 17 into Supplementary Equation 15, we obtain

$$\frac{d\Delta v_{\mathrm{g}}}{dt} = -KB\Delta z . \tag{18}$$

In addition to these long-range forces described above, solitons also experience a gain filtering effect in the EDFA, leading to frequency rolling forward the frequency of the maximum gain, which can be described as[4,22,23]

$$\frac{d\Delta\omega}{dt} = -\Gamma\Delta\omega \ , \tag{21}$$

Using Supplementary Equation 16 and Supplementary Equation 21, we then obtain

$$\frac{d\Delta v_{\mathrm{g}}}{dt} = -\Gamma\Delta v_{\mathrm{g}} \tag{22}$$

Next, we introduce a random driving term $S_{\Delta\omega}$ to describe the white noise, which results from the amplified spontaneous emission (ASE) of the EDFA and can be written as[22]

$$\begin{cases} \langle S_{\Delta\omega} \rangle = 0 \ , \\ \langle S_{\Delta\omega}(t) S_{\Delta\omega}(t') \rangle = N_{\Delta\omega}\delta(t-t') \ , \end{cases} \tag{23}$$

where the bracket $\langle ... \rangle$ denotes an ensemble average, $N_{\Delta\omega}$ denotes the noise level, and the delta function indicates that noise at different time is uncorrelated[22].

In sum, the coupled equations that describe the motion of the second soliton in the vicinity of the equilibrium position are given by

$$\begin{cases} \dfrac{d\Delta z}{dt} = \Delta v_{\mathrm{g}} \ , \\ \dfrac{d\Delta v_{\mathrm{g}}}{dt} = -KB\Delta z - \Gamma\Delta v_{\mathrm{g}} + S_{\Delta\omega} \ . \end{cases} \tag{24}$$

For simplicity, we replace $(\Delta z, \Delta v_{\mathrm{g}})$ with (z, v), we obtain the Langevin equations in the presence of a harmonic potential[24,25]. The mean square deviation of z is

$$\left\langle \left| z(t) - z_0 \right|^2 \right\rangle = \frac{N_{\Delta\omega}}{2\Gamma KB} \ . \tag{25}$$

The presence of the harmonic potential implies that the mean square deviation of the relative pulse position (timing jitter of pulse spacing) does not grow with time (see Supplementary Figure 5b). The experimental results demonstrated in Fig. 2b in the main text have confirmed this feature of the soliton supramolecule.

In contrast, when the spring effect (harmonic potential, i.e. the term $-KB\Delta z$) is absent, this equation would degrade into the random-walk equations that describe for example the Brownian motion of a particle in a liquid. The mean square deviation of the particle position $z(t)$ can be expressed using the well-known diffusion equation as

$$\left\langle \left| z(t) - z_0 \right|^2 \right\rangle = \frac{N_{\Delta\omega}}{\Gamma^2} t \ . \tag{28}$$

In this case the mean square deviation of the relative pulse position would increase linearly with time (see Supplementary Figure 5a), corresponding to the well-known Gordon-Haus jitter in soliton communications systems[23].

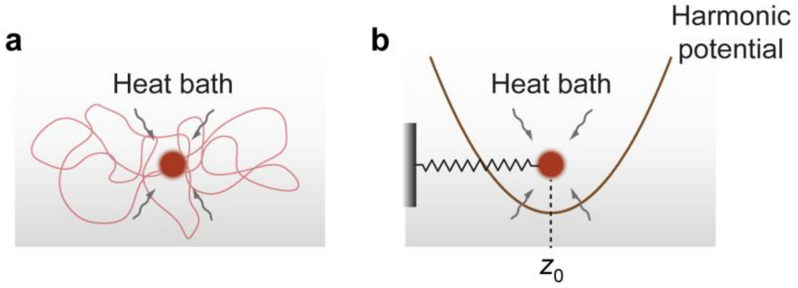

Supplementary Figure 5 | Random walk of a particle, a. When the particle is in the heat bath, with a mean square deviation growing linearly with time. **b,** when the particle is trapped within a harmonic potential, whose displacement has a constant mean square deviation despite continuous perturbations of the heat bath.

Supplementary Note 4: Long-term preservation of soliton supramolecules

In the experiments we obtained a variety of soliton supramolecules with different solitons numbers and structures, all of which exhibited long-term stability. The full plot (over one complete cavity round trip) of a typical supramolecular assembly of optical soliton is plotted in Supplementary Figure 6a, part of which is also shown as Fig. 2a in the main text.

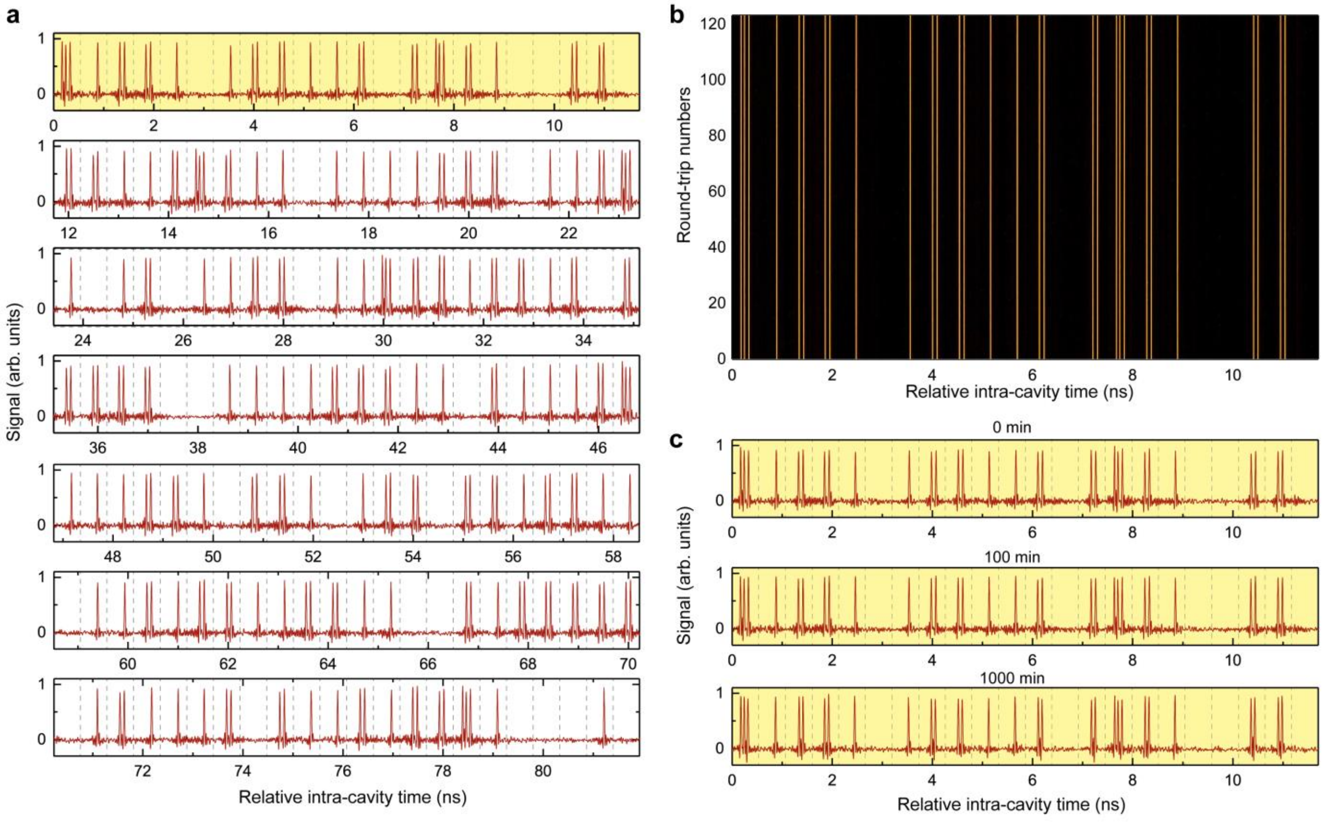

Supplementary Figure 6 | A typical soliton supramolecule. a, The time-domain trace recorded over one cavity round trip using oscilloscope. **b**, The round-trip plot of the same soliton supramolecule over 123 consecutive round trips (~10 μs). **c**, The highlighted part in (a) is recorded at 0 min, 100 min, and 1000 min after the soliton supramolecule is generated.

This soliton supramolecule is composed of null-soliton, single-soliton, double-soliton, and triple-soliton units in the time-slots of the optomechanical lattice, and the entire pattern has been observed to be perfectly preserved over more than one week without any obvious degradation in signal-to-noise ratio

(see Supplementary Figure 6b and c). In the experiments the laser output is recorded at 0 min, 100 min, and 1000 min after the soliton supramolecule is generated. The result (only the first 22 units highlighted in Supplementary Figure 6a are shown due to the limited space) is plotted in Supplementary Figure 6c, demonstrating error-free preservation of the supramolecular pattern over 1000 minutes.

As shown in Fig. 2b in the main text, we observe a slight change of internal spacing at 1000 min compared to the previous two measurements. We attribute this change to long-term mechanical drifts of the FPCs in the cavity which could lead to a slight change of the laser working point[4,7]. Nevertheless, the soliton supramolecule is able to respond to these slow environmental drifts, stably preserving basic pattern of the supramolecular structure.

Supplementary Note 5: Uncorrelated phase relations

Conventional soliton molecules that rely on direct overlap of the tails of individual solitons, feature not only a close inner spacing, but also correlated carrier phases between the strongly-bound solitons[10–13]. By contrast, the soliton supramolecules that we studied in this paper are bound by weak, long-range forces These forces bind a large number of solitons with much wider spacing, and the carrier phases of the solitons are no longer correlated. In this Note, we provide direct experimental evidence of the uncorrelated phases.

In order to directly read the carrier-phase information of the self-assembled solitons, we use a narrow-linewidth (2 kHz) CW light source (as a local oscillator) to heterodyne with the supramolecular soliton sequence output from the optoacoustically mode-locked fibre laser (see Supplementary Figure 7a). We use a 50:50 fibre coupler to combine the mode-locked laser output and the CW light. Two FPCs are inserted to adjust the polarization states for two arms of light path in order to maximize the interferometric contrast. The beating signal out of the coupler is recorded using a fast oscilloscope and an optical spectrum analyzer. When the central wavelength of both lasers are at 1550 nm (see Supplementary Figure 7b), the optical comb structure of the mode-locked laser is downshifted to the radio-frequency range (see Supplementary Figure 7c). We then acquire the phase information of the laser solitons that has been translated into RF amplitude modulations using the photodiode and the oscilloscope. The soliton supramolecule measured in the experiments before turning on the CW light source is partially shown in Supplementary Figure 7d, and is composed of single-, double-, and triple-soliton units. When the CW light source is turned on, and the FPCs are carefully adjusted, we obtaine the heterodyne signal (see Supplementary Figure 7e) showing that the pulse amplitudes are strongly modulated due to the interference with the CW light. We acquire a trace of the 20-μs-long heterodyne signal, which corresponds to ~200 consecutive cavity round trips. When the heterodyne signal is plotted in parallel against the round-trip number, as shown in Supplementary Figure 7f, each individual soliton exhibits a clear sinusoidal beating with a period matching the beat frequency shown in Supplementary Figure 7c. These results indicate that both the amplitude and the phase of each soliton are preserved over many cavity round trips. However, no obvious phase relation among the beating patterns is observed, indicating uncorrelated carrier phases of these solitons inside the self-assembled supramolecular structure.

The uncorrelated phases can also be indirectly inferred from the optical spectra of the soliton supramolecules. As shown in Supplementary Figure 8g–i and 9e–g, the central parts of these soliton laser spectra with the highest power densities exhibit smooth profiles with no fringe pattern, indicating further that the solitons involved in the supramolecular assembly are not locked in phase.

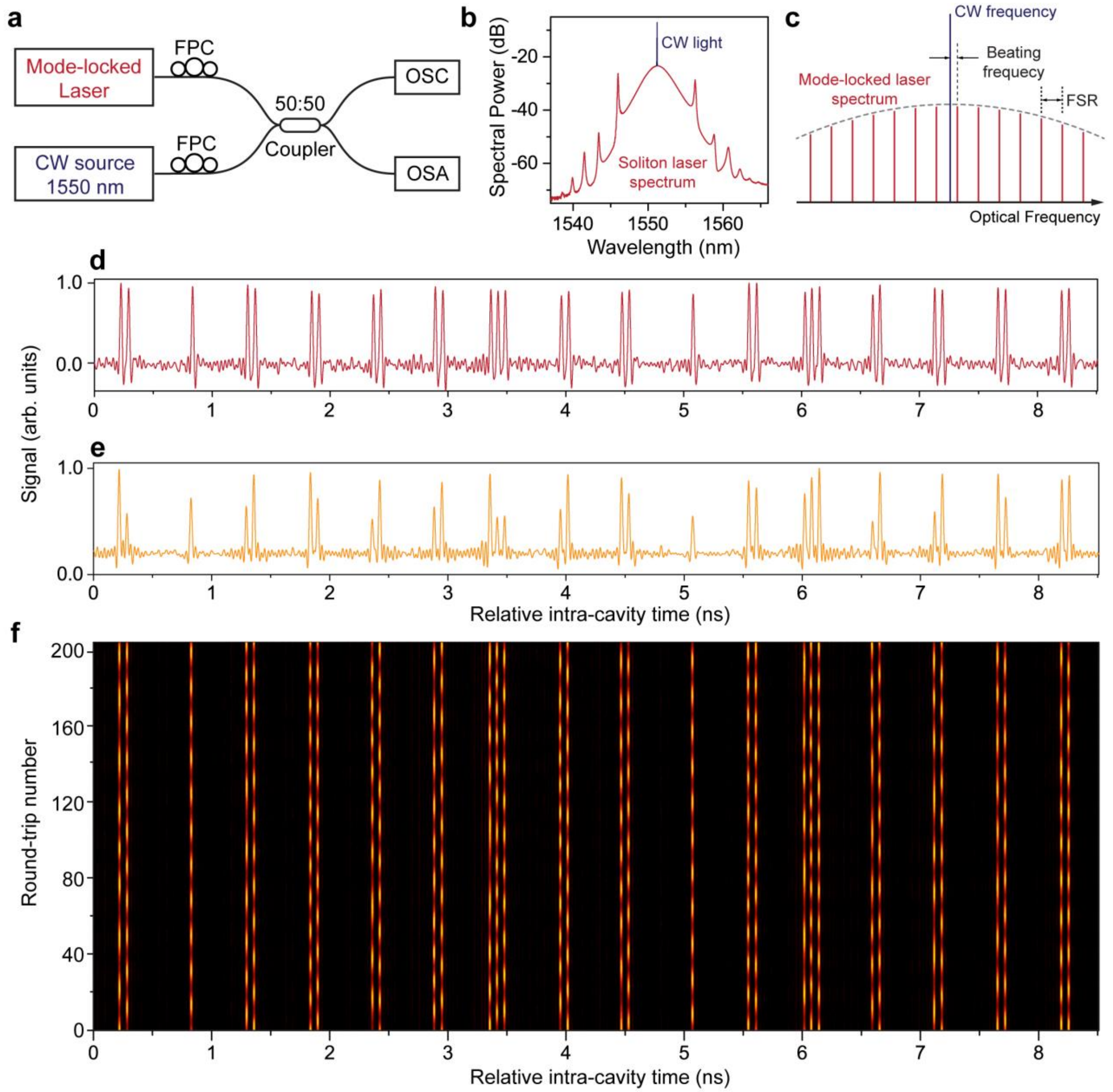

Supplementary Figure 7 | Heterodyne experiment of soliton supramolecules. **a**, Sketch of the experimental set-up. **b**, The optical spectrum of the combined signal from both the mode-locked fibre laser and the CW light source. **c**, Conceptual sketch of the frequency comb structure of the mode-locked laser and the single-frequency local oscillator. **d**, Part of the soliton supramolecule sequence without heterodyning. **e**, The same sequence that are strongly modulated after turning on the local oscillator. **f**. The 20-μs-long heterodyne signal (plotted in parallel over ~200 cavity round trips) exhibits uncorrelated sinusoidal beating patterns, indicating uncorrelated phases of the solitons in the self-assemble structure.

Supplementary Note 6: Uniform soliton supramolecules

In the experiments we are able to reproducibly generate a supramolecular assembly of solitons with homogeneous patterns, including all-double-soliton (ADS) and all-triple-soliton (ATS) supramolecules. Some of the fundamental properties of the supramolecular assembly of optical solitons can best be demonstrated using these uniform patterns.

All-double-soliton supramolecules

Supplementary Figure 8a shows the time-domain trace of the ADS supramolecule recorded using the OSC over one cavity round trip (with only the starting and ending parts of the pulse train). As we increase the recording time, we plot the time-domain trace over many cavity round trips (the round-trip time is ~85 ns) in the persistence mode as shown in Supplementary Figure 8b, which exhibits a perfect preservation of those double-soliton units in the optomechanical lattice. We also illustrate the experimental recording in a third way as shown in Supplementary Figure 8c, in which we separately plot the time-domain trace within each time-slot of the lattice (the period of the lattice is ~0.532 ns) along the ordinate, and we repeat this plot for the 160 consecutive time-slots within a cavity round trip along the abscissa. These plots exhibit the relative positions of the double-soliton units in the optomechanical lattice. Supplementary Figure 8d shows the same pulse train recorded with the persistence mode over a single time-slot span, from which we can clearly see the characteristic inter-soliton spacing of 90 ps which is the identical in all the time-slots. The FFT spectrum of the time-domain trace shown in Supplementary Figure 8e exhibits a sinusoidal envelope with a period of 11.1 GHz, which agrees well with the 90-ps pulse spacing. The pulse duration measured using the autocorrelation trace is only 670 fs (see Supplementary Figure 8f), while the soliton-soliton spacing in each unit is more than one hundred times longer (90 ps).

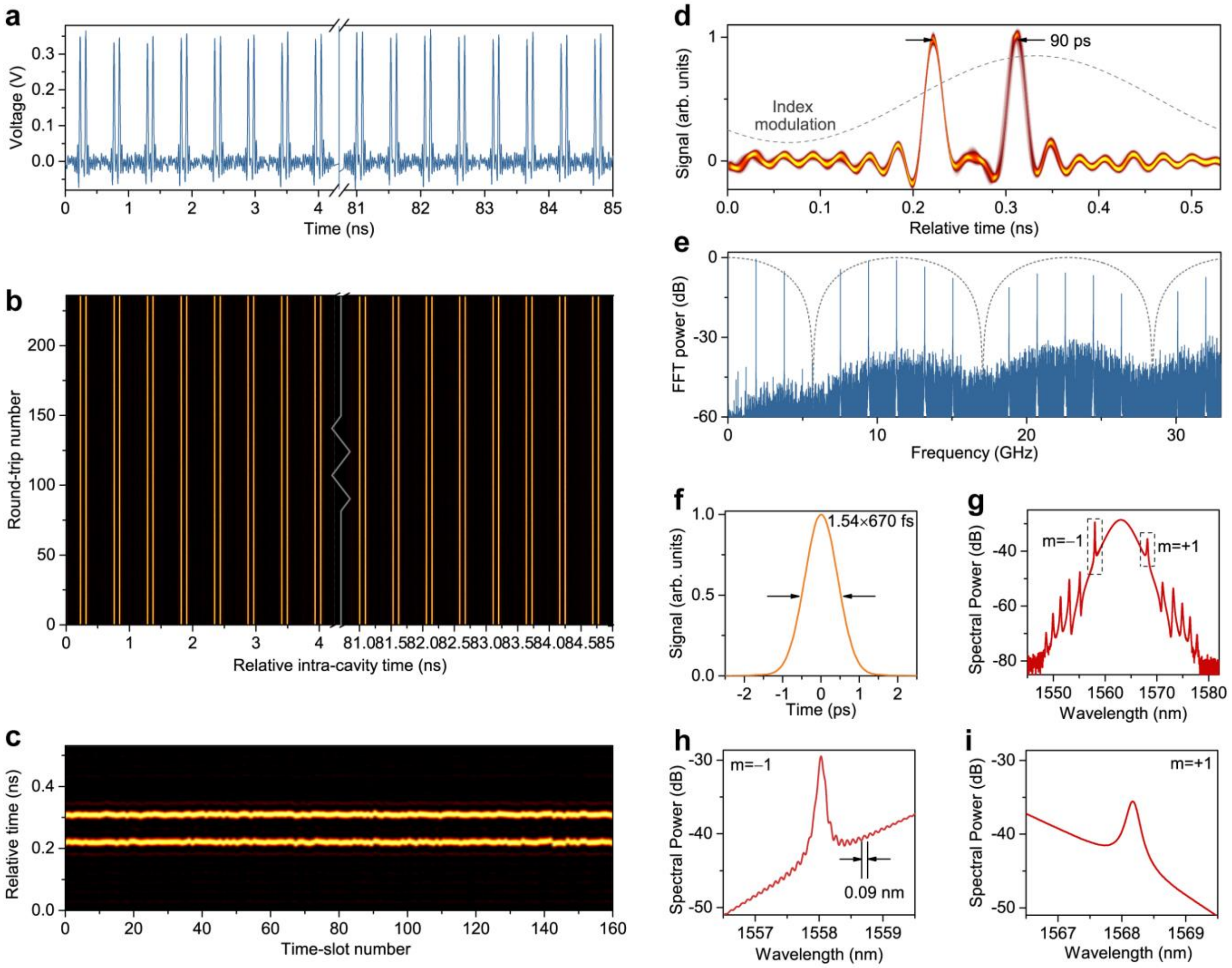

Supplementary Figure 8 | Experimental recordings of an all-double-soliton supramolecule. a, Time-domain trace of the supramolecule recorded using the OSC. Due to the limit of figure size, the trace is plotted over only 8 ns (the cavity round-trip time is ~85 ns). **b**, A plot of a longer time-domain trace over 230 cavity round trips in the persistence mode. **c**, The time-domain traces in the 160 time-slots of the lattice plotted in parallel. The vertical axis corresponds to the relative time in each time-slot (the period of lattice is ~0.531 ns), while the horizontal axis corresponds to the 160 consecutive time- slots. **d**, The time-domain trace of the ADS supramolecule recorded with the persistence mode, exhibiting a stable 90-ps internal spacing. **e**, The FFT power spectrum of the time-domain trace with a sinusoidal envelope (dashed black line). **f**, The autocorrelation trace of the ADS supramolecule. **g**, The optical spectrum of the ADS supramolecule, with the dominant sideband marked in the dashed box (m = –1 order sideband at shorter wavelength and m = +1 order at longer wavelength). **h**, Expanded view of the spectrum in (g) at the dominant sideband (m = –1 order). **i**, Expanded view of the spectrum in (g) at the other sideband (m = +1 order).

In Supplementary Figure 8g, we show the optical spectrum of the ADS supramolecule, featuring a sech^2-shaped profile and characteristic Kelly sidebands[17–19]. Some spectral fringes are observed only in the vicinity of the dominant (m = –1 order) sideband, indicating stable phase locking between the dominant dispersive wave and the soliton (see Supplementary Figure 8h), while near the m = +1 sideband we cannot observe an interferometric fringe (see Supplementary Figure 8i). The interferometric fringes in the vicinity of the m = –1 sideband have a period of 0.09 nm (or 11.1 GHz), agreeing well with the 90-ps internal soliton spacing in each time-slot. All of these features agree well with the theoretical model presented in Supplementary Note 2.

All-triple-soliton supramolecules

By carefully increasing the laser pump power and adjusting the intra-cavity FPCs, we obtain soliton supramolecules with homogeneous all-triple-soliton (ATS) units. Like in Supplementary Figure 8c, we plot the time-domain trace of the ATS supramolecule over one cavity round trip. As shown in Supplementary Figure 9a, the consecutive time-slots are plotted in parallel, in order to show that all the triple-soliton units have the same inter-soliton spacing and relative positions within the optomechanical lattice. In Supplementary Figure 9b, we also show the persistence-mode recording over one time-slot span, which exhibits tiny variations of the inter-soliton spacing. The measured internal spacing between consecutive solitons is ~76 ps (between first and second solitons) and ~71 ps (between second and third solitons). The FFT power spectrum of the ATS supramolecule (see Supplementary Figure 9c) exhibits a modulated envelope corresponding to the inter-soliton spacing inside the triple-soliton units. In Supplementary Figure 9d, we show the autocorrelation trace of the ATS supramolecule, which corresponds to an individual soliton duration of 690 fs. In Supplementary Figure 9e, we show the optical spectrum of the ATS supramolecule, in which spectral fringes are visible in the vicinity of the dominant sideband (see Supplementary Figure 9f), with a fringe period agreeing well with the internal spacing between the solitons. The fringe contrast is weak, because two different fringe periods coexist, corresponding to two characteristic inter-spacing values in the triple-soliton units. As a consequence, the interferometric signal during the OSA measurement is blurred. As expected, the sideband at m = +1 order exhibits no fringe (see Supplementary Figure 9g).

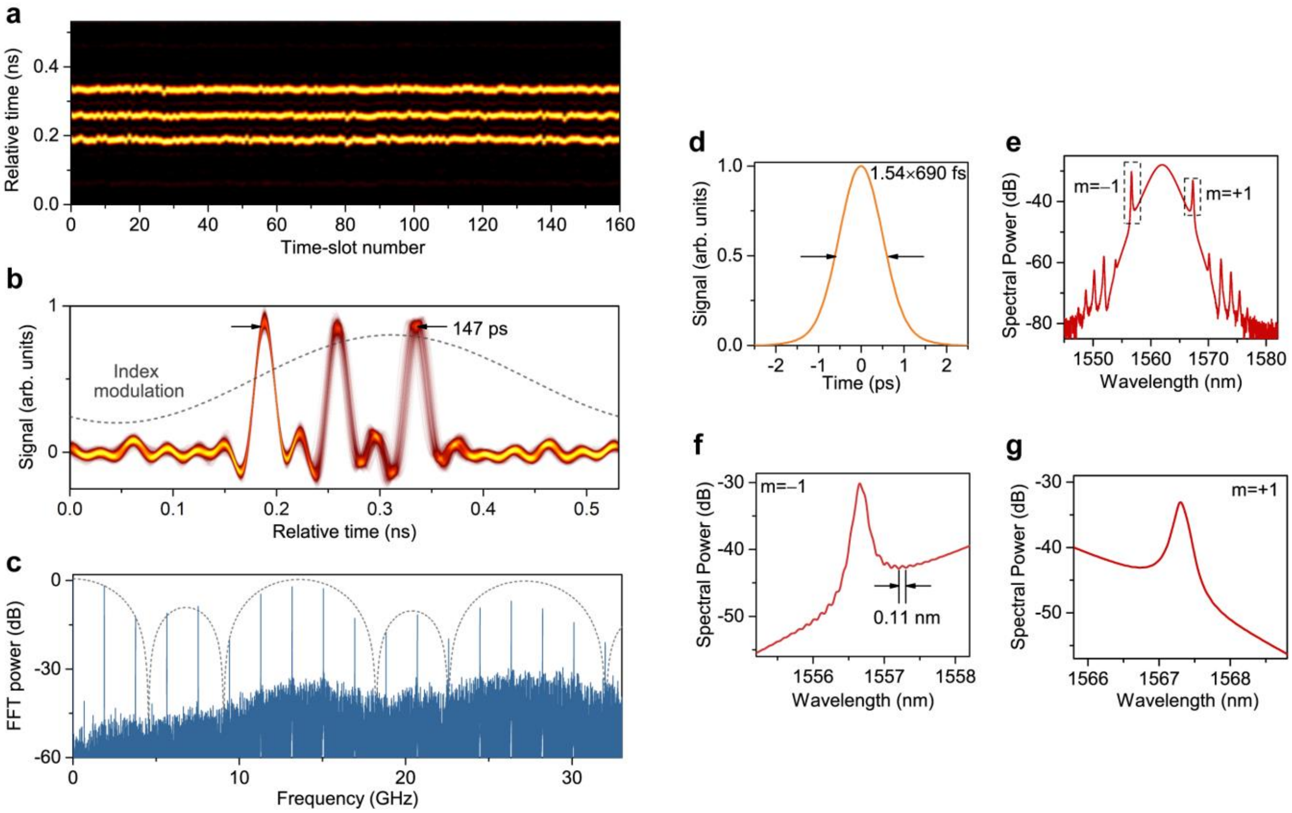

Supplementary Figure 9 | Experimental recordings of an all-triple-soliton supramolecule. **a**, The time-domain trace of the ATS supramolecule over one cavity round trip, with all the 160 time-slots plotted in parallel. **b**, The same time-domain trace plotted with the persistence mode over one time-slot span. **c**, The FFT power spectrum of the ATS supramolecule. **d**, The autocorrelation trace. **e**, The optical spectrum. **f**, Expanded view of the dominant (m=–1 order) sideband on the optical spectrum. **g**, Expanded view of the m = +1 order sideband.

The experimental results, together with these from the ADS supramolecule, confirm the fact that the repulsive force between the solitons is related to the dominant Kelly sideband, and fixed phase relations between the dispersive waves and perturbed solitons are necessary to achieving stable long-range binding of solitons, as predicted in Supplementary Note 2.

Generation of quadruple-soliton units in the soliton supramolecule requires a higher pump power. In our experiments we are only able to fill half of the time-slots with quadruple-soliton units while the others are only filled with triple-soliton units, due to the limited (~1.5 W) pump power. The time-domain trace of this soliton supramolecule is partially shown in Supplementary Figure 10a. This pattern could also be stably preserved over 20 minutes (see Supplementary Figure 10b), even though the quadruple-soliton units are slightly unstable compared to the double- and triple-soliton units. We attribute the decreased stability to a weaker trapping potential that makes the fourth soliton in the quadruple-soliton unit more vulnerable to noise perturbations, leading to a larger timing jitter (see also Fig. 2e in the main text).

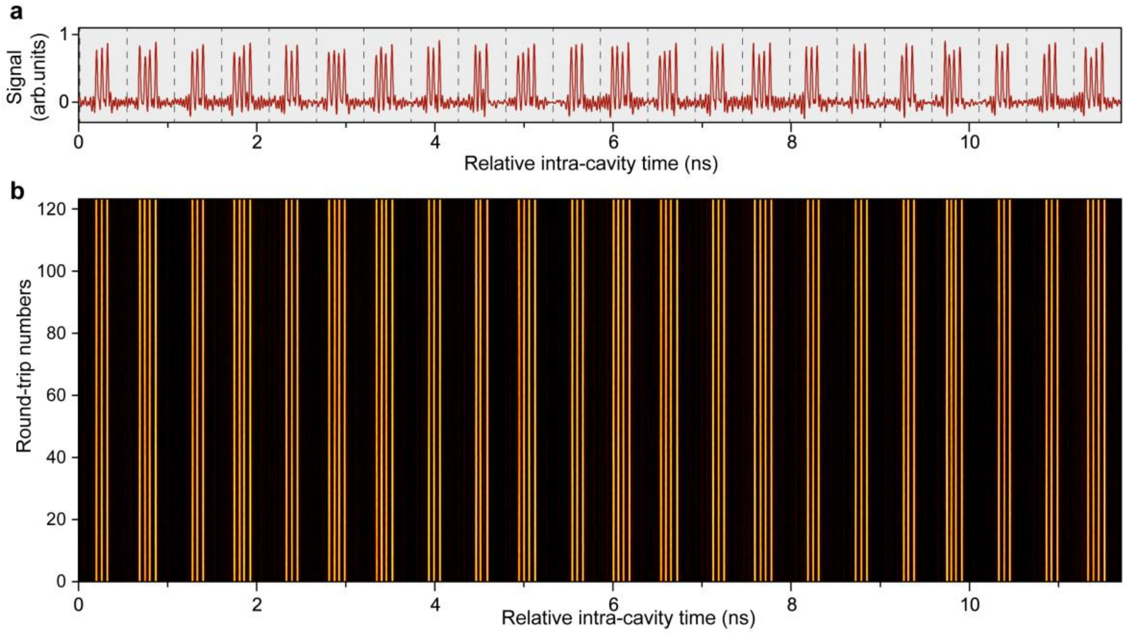

Supplementary Figure 10 | a, The time-domain trace of the soliton supramolecule that contains triple- and quadrupole-soliton units (only 23 out of 160 time-slots are plotted). **b**, The persistence-mode plot of the same time-domain trace over 123 cavity round trips (over ~10 μs).

The maximum number of solitons that can be trapped within each supramolecular unit is limited roughly by the ratio between the acoustic period and the inter-soliton spacing, and the total number of units in the supramolecular structure is equal to the ratio of the cavity round-trip time to the acoustic period. The highest number of units reached in the experiments is more than one thousand. Actually, to accommodate more than two solitons in each unit, the strengths of the long-range forces needed to be tailored down in order to shift the trapping potentials for additional solitons closer to the first soliton, making the whole unit more compact. Facilitated by controlling technique we implement in the laser cavity, such shifting of trapping potentials can be realized by enhancing the acoustic amplitude or by decreasing the dispersive wave amplitude. Currently four solitons at most can be bound stably at long-range within each unit, limited by the sub-ns acoustic cycle corresponding to the GHz-rate acoustic resonance. More solitons can be bound in a single unit by increase the acoustic period and/or by combining both long-range and short-range binding (e.g. soliton molecules, as illustrated in Fig. 3 in main text, as well in Supplementary Note 8 below). For fixed cavity length the maximum total number of solitons in the entire cavity that can be self-assembled is limited by the maximum single-pass EDFA gain, which can be as high as several thousand. The potential optical information storage capacity is thus more than one thousand bits, and can

further expand by incorporating diverse solitons molecules as fundamental building blocks that could go beyond binary coding strategy[26].

Supplementary Note 7: Directions of the repulsive forces

This Note aims to elucidate the full picture of the possible time-domain relation of the multiple solitons bound within one time-slot of the optomechanical lattice. Previously we only illustrated one possibility of the power distributions among different Kelly-sidebands, in which the $m = -1$ order sideband has much higher intensity than all the others. This dominant sideband could be induced by the uneven gain profile in the EDFA[20] as well as the higher-order dispersion of the laser cavity[19]. When we assume that the two first-order Kelly-sidebands symmetrically located on the soliton spectrum so that the absolute values of their deviations from the soliton central frequency are equal, the uneven gain profile in the EDFA leads to different gains for these two Kelly-sidebands, leading to an intensity difference between them. On the other hand, higher-order dispersion in the cavity would result in an asymmetric distribution of the two first-order sidebands on the soliton spectrum, leading to a difference in energy coupling from solitons[16,19]. We adjust the relative intensities of the two first-order sidebands by carefully adjusting the gain in the EDFA by changing the cavity loss and by adjusting the high-order dispersion of the laser cavity through inserting dispersion-compensation fibre into the cavity.

With different relative intensities of the two sidebands, the configuration of the repulsive forces induced by the dispersive wave perturbations changed significantly. We create hybrid soliton supramolecules in which the single-soliton and double-soliton units coexist in the optomechanical lattice. Using a time-slot plot similar to that shown in Supplementary Figure 8c, we obtain more information about the direction of the long-range repulsion forces between the two solitons. We show three typical results for different relative intensities of the two first-order ($m = \pm1$) sidebands.

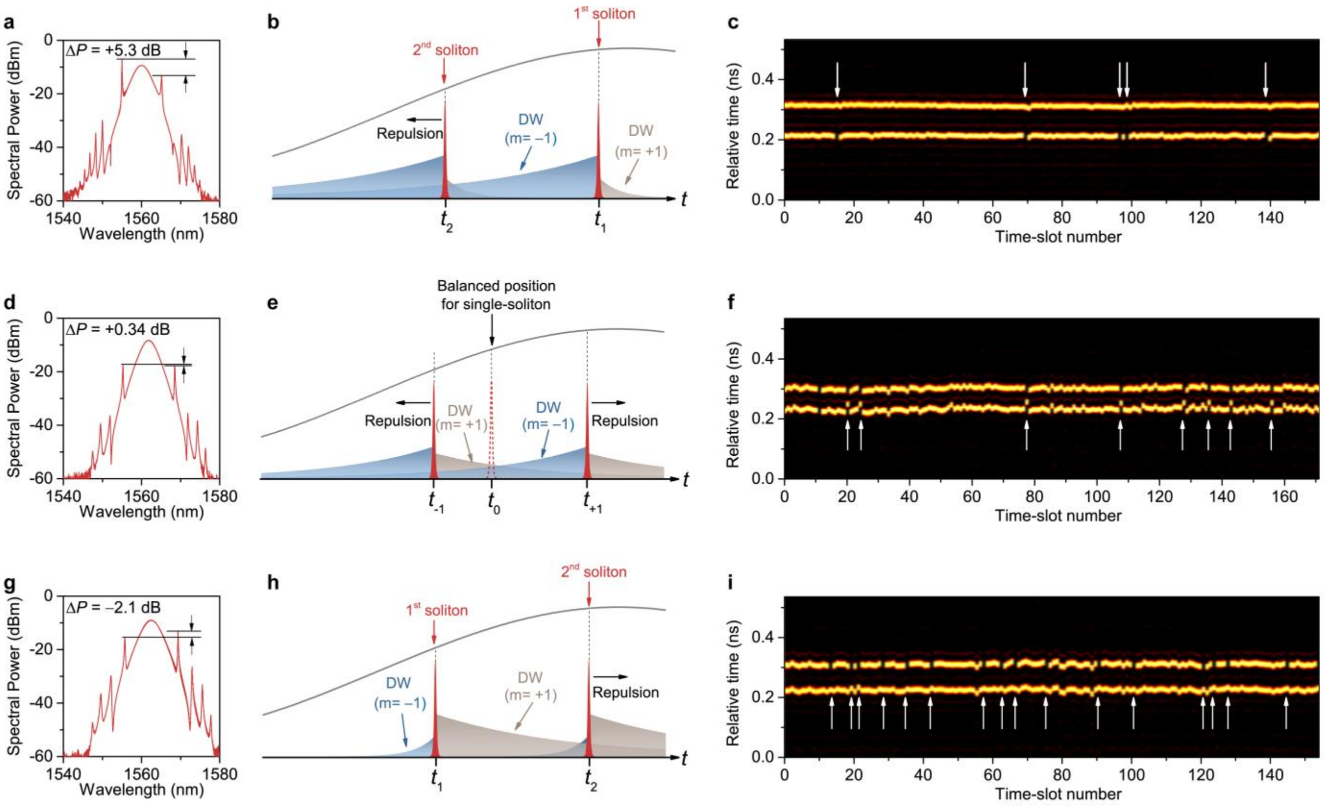

Supplementary Figure 11 | Three cases of the repulsive force due to dispersive wave perturbations. a, The case that the $m = -1$ order sideband dominates. **b**, The dispersive waves shed by the soliton are located later in time (set as the first soliton) perturbs the soliton located earlier in time, leading to an effective force of repulsion. **c**, The hybrid soliton supramolecule with several single-soliton units. The positions of those single-soliton units are marked by the white arrows (the upper row of pulses). **d** – **f**, The two first-order ($m = \pm 1$) sidebands have comparable intensities in the second case. Both of these sidebands (dispersive waves) will contribute a force of repulsion between the two solitons and push the two solitons in the double-soliton unit away from a balanced position. **g** – **i**, When the $m = +1$ sideband dominates, the first soliton is earlier in time. The dispersive wave shed from this soliton travels slower than the soliton, and perturbs the second soliton that is located later in time. We then find that the solitons in single-soliton units that are located at the same relative position as the first soliton in the double-soliton unit (the lower row of pulses in **i**).

In the first case, as shown in Supplementary Figure 11a, when the shorter-wavelength ($m = -1$ order) sideband dominates with a higher intensity (ΔP = +5.3 dB), we set the soliton located later in time as the reference (first) soliton (see Supplementary Figure 11b). In this case, the force of repulsion in the double-soliton unit is induced by perturbations of the dispersive wave shed from the first soliton, while the $m = +1$ order dispersive wave shed from the second soliton has a much weaker intensity. When a single soliton is trapped in one time-slot of the lattice, it locates at the same position as that of the first soliton in the double-soliton unit, since neither of them experiences significant perturbations from dispersive waves. As shown in Supplementary Figure 11c, we record a hybrid soliton supramolecule in which several time-slots has only trapped one soliton. In these single-soliton units (marked by the white arrows), the solitons are always located at the upper row, which corresponds to the later (the first soliton) position in Supplementary Figure 11b. In the second case, as shown in Supplementary Figure 11d, comparable intensities of the two ($m = \pm 1$ orders) sidebands (ΔP = +0.34 dB) lead to two comparable forces of repulsion experienced by both of the two solitons in one time-slot (Supplementary Figure 11e), which push them away from the balanced position as shown in Supplementary Figure 11f. The third case is that the longer-wavelength ($m = +1$ order) sideband is dominant with ΔP = –2.1 dB (see Supplementary Figure 11g). In contrast to the first case shown in Supplementary Figure 11a–c, in this case we use the

soliton earlier in time as the reference (first) soliton, since in this case the dispersive wave shed from this soliton earlier in time results in a repulsive force between the two solitons in one time-slot. The $m = +1$ order dispersive wave shed from the first soliton travels more slowly and perturbs the second soliton located later in time (Supplementary Figure 11h). As a consequence, in single-soliton units the soliton is always located at the lower row (see Supplementary Figure 11i) which corresponds to the earlier soliton position in Supplementary Figure 11h.

We emphasize that in most of the experiments the $m = -1$ order sideband at the shorter-wavelength has the highest intensity and dominated the repulsive force as shown in Supplementary Figure 11a. In some experiments, however, we obtain an optical spectrum with almost uniform two ($m = \pm 1$ order) sidebands (see Supplementary Figure 11d) or with a higher intensity at the $m = +1$ order sideband (see Supplementary Figure 11) through carefully adjusting the laser cavity loss and simultaneously inserting some dispersion-compensation fibre into the laser cavity. The experimental results shown in Supplementary Figure 11 did not alter the physical picture of the soliton supramolecule whose formation relies on long-range, inter-soliton interactions. Moreover, the results that we show in Supplementary Figure 11 are consistent with the theoretical prediction in Supplementary Note 2. We conclude that supramolecular assemblies of fibre laser solitons can be obtained within a broad range of system parameters.

Supplementary Note 8: Details of elementary diversity

Soliton supramolecules consisting of phase-locked soliton-pair molecules

Soliton supramolecule can incorporate XPM-based soliton molecules with a small inner-spacing and specific phase-relations as fundamental building blocks. One type of soliton molecule that can be incorporated in the supramolecular assembly is a phase-locked soliton pair with a phase difference of π. We are able to reproducibly generate uniform soliton supramolecules in which each time-slot of the optomechanical lattice is filled with a phase-locked soliton-pair molecule and a single soliton. The time-domain trace recorded by the oscilloscope with the persistence mode is shown in Supplementary Figure 12a (with a one-time-slot span), exhibiting two pulsed signals with an 80-ps internal spacing, in which the later pulse has twice the amplitude of the earlier one. Due to the relatively slow response time of the oscilloscope (~ 20 ps), a soliton-pair molecule with an inner spacing of a few ps can only be detected as a single pulse with twice the energy. The optical spectrum of this soliton supramolecule, as shown in Supplementary Figure 12b, exhibits strong interferometric fringes over the entire spectrum with a period of 1.83 nm. The dip in the centre of the spectrum implies a phase difference of $\sim\pi$ between the two solitons in the pair. The long-range repulsive force exerted by the soliton-pair upon the single soliton in the same time-slot leads to local fringes in the dominant Kelly sideband, as shown in the Supplementary Figure 12c. The 0.12 nm fringe period agrees well with 80-ps internal spacing, indicating a similar binding mechanism in the case of the ADS supramolecule shown in Supplementary Figure 8, although the fringes have a lower contrast due to the weaker intensity of the Kelly sideband in this case. The entire soliton supramolecule structure over one cavity round trip is plotted against the time-slot number in Supplementary Figure 12d, exhibiting the same relative positions of the two building blocks in each time-slot. Using the TS-DFT method to stretch this soliton supramolecule over a 2-km-long SMF-28 fibre, we observe that the DFT signal of the phase-locked soliton pair exhibits strong interferometric fringes, while the DFT signal of the single soliton exhibits no obvious fringe (see Supplementary Figure 12e).

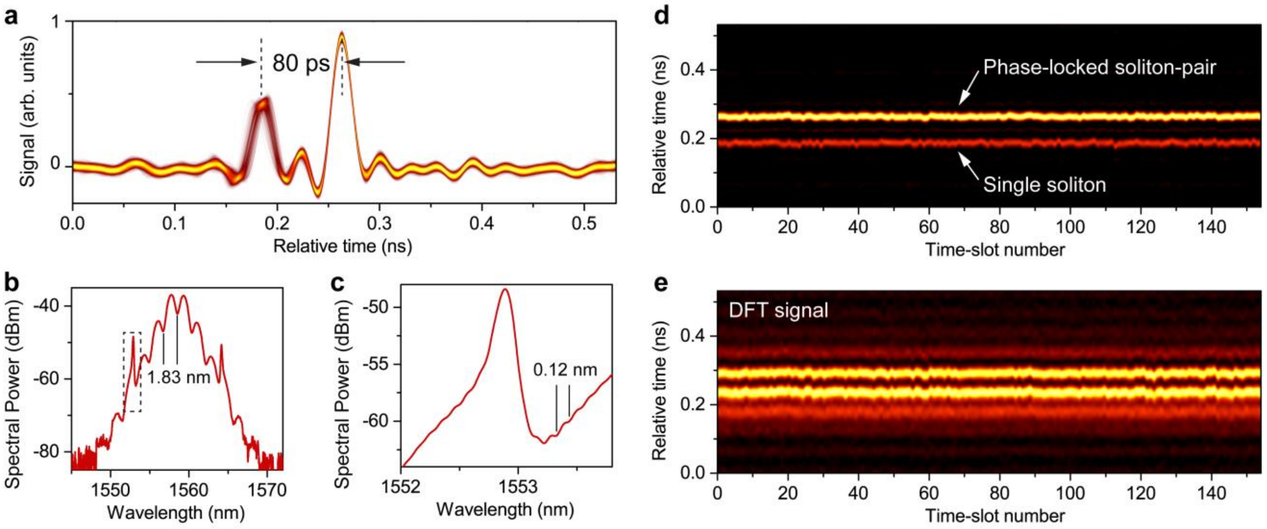

Supplementary Figure 12 | Uniform soliton supramolecule including single solitons and phase-locked soliton pairs. a, The time-domain trace of the uniform supramolecule recorded under persistence mode, which includes in each unit a single soliton and a phase-locked soliton-pair, with stable spacing of 80 ps between them. **b**, The optical spectrum of this soliton supramolecule. Strong spectral fringes with a period of 1.83 nm appear due to the presence of phase-locked soliton-pairs. **c**, Expanded view of the dominant Kelly sideband, where local fringes have been observed with a period of 0.12 nm, matching well with the 80 ps internal spacing. **d**, The entire soliton supramolecule composed of 154 time-slots (over one round trip) plotted against time-slot number. **e**, The DFT signal of this soliton supramolecule.

In Supplementary Figure 13a, we plot the entire structure of the soliton supramolecule that we already partially showed in Fig. 3a – c in the main text, as a function of the time-slot number, and we show the DFT signal in Supplementary Figure 13b. Similar to the case shown in Supplementary Figure 11a – c, a repulsive force is exerted in one time-slot of the optomechanical lattice by the first element, either a single soliton or soliton-pair, on the second element that earlier in time. However the presence of the second component does not affect the position of the first component. This lack of reciprocity leads empty positions that appear at earlier positions in each time-slot (in the lower row in Supplementary Figure 13a).

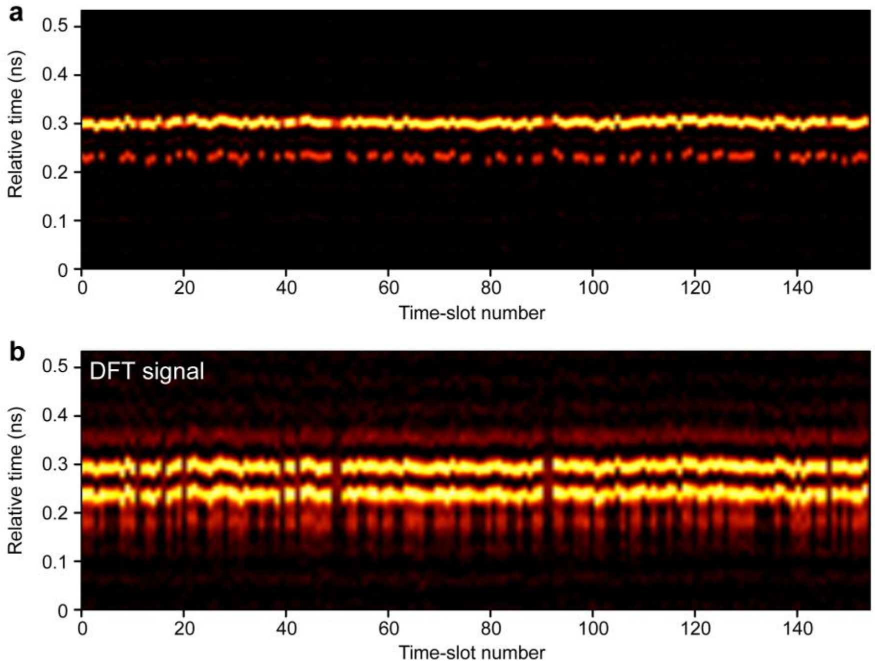

Supplementary Figure 13 | Hybrid soliton supramolecular structure composed by both single solitons and phase-locked soliton pairs (time-domain trace partially shown in Fig. 3a – c in the main text). **a**, The real-time trace of the entire structure plotted against the time-slot number. **b**, The DFT-signal of the soliton supramolecule, showing strong fringes in the time-slots that consist of stable soliton pairs.

Soliton supramolecules consisting of phase-drifting soliton-pair molecules

In addition to soliton pairs with fixed phase differences, we also observe in some soliton supramolecules a special type of soliton pair, in which the phase difference between the two strongly-bound solitons diverges almost quasi-linearly during propagation. This continuous divergence of the phase difference between the two soliton in a pair has been previously reported, and the soliton pair is referred so as a phase-drifting soliton pair[27]. The drifting phase difference possibly stems from slightly different peak intensities of the two solitons, which leads to different rates of nonlinear phase accumulation during their propagation. In Fig. 3f in the main text we illustrate part of a soliton supramolecule composed of phase-drifting soliton pairs and single solitons. A stable internal spacing of 40 ps is observed between the phase-drifting soliton pair and the single soliton within one time-slot. We first analyzed the property of the phase-drifting soliton-pair using the DFT signal. As described in reference [13], we can retrieve the phase differences and inner-spacing of the soliton pairs from the fringe patterns of the DFT signal. For a soliton pair with an inner spacing τ and a phase difference $\Delta\varphi$, the electric field of the soliton pair can be written as

$$E(t) = \mathrm{Re}\left\{\left[E_1(t) + E_1(t+\tau)\exp(i\Delta\varphi_0)\right]\exp(i\omega_0 t)\right\}, \tag{29}$$

where $E_1(t)$ is the profile and ω_0 is the carrier frequency of a single soliton. We assume that the two solitons in the pair have the same profile, in which case the interferogram $S(\omega)$ of the soliton pair would be[13]

$$S(\omega-\omega_0) \propto \left|E(\omega-\omega_0)\right|^2\left[1+\cos\left((\omega-\omega_0)\tau + \Delta\varphi\right)\right], \tag{30}$$

where $E(\omega)$ is the spectral profile of a single soliton. In practice, we fit the time-domain DFT signal obtained in the experiments using the function

$$S(t) = A\operatorname{sech}^2\left(\frac{t-t_0}{\Delta T}\right)\left[1+\cos\left(\frac{2\pi}{\Delta t}(t-t_0)+\Delta\varphi\right)\right]+B \ , \tag{31}$$

where A, B, and t_0 are the fitting parameters related to the envelope amplitude and offset in the selected coordinate. Assuming that the fibre used in the DFT set-up has a dispersion β_2 and length L, then ΔT is linearly related to the soliton spectral bandwidth $\Delta\omega$ by the relation $\Delta T \propto \beta_2 \Delta\omega L$, in which the constant of proportionality depends on the definition of bandwidth. The period of the cosine modulation Δt relates to the inner spacing τ of the soliton pair as[13]

$$\Delta t = \frac{2\pi\beta_2 L}{\tau} \ . \tag{32}$$

The parameters τ and $\Delta\varphi$ that we retrieve on consecutive cavity round trips are illustrated in Supplementary Figure 14. Using the DFT signal acquired in the experiments, as shown in Supplementary Figure 14a, corresponding to the signal in the third time-slot in Fig. 3f from left in the main text, we can obtain the phase difference $\Delta\varphi$ (see Supplementary Figure 14b) and the inner spacing in the soliton pair (see Supplementary Figure 14c) using Supplementary Equations 31 and 32. We find that the phase difference between the two solitons decreased approximately linearly over consecutive cavity round trips, accompanied by a weak sinusoidal modulation (see Supplementary Figure 14b). The rate of the phase drift is estimated to be -0.06π per cavity round trip (a change of -2π after each ~33 round trips), and the weak sinusoidal modulation is observed at the same frequency. Therefore the phase difference can be approximately described as[27]

$$\Delta\varphi(z) = -z + A_\varphi \sin(z+\phi_0) + \Delta\varphi_0 \, , \tag{33}$$

where $z = 0.06\pi n$ (n is the round-trip number), $A_\varphi = 0.505$, and the example we illustrate here gives the initial phase value $\Delta\varphi_0 = -0.24\pi$ and $\phi_0 = 0.36\pi$. This analytical fit is also plotted in Supplementary Figure 14b, and is deliberately shifted by 2π for clarity. We also observe some supramolecular structures in which phase-drifting soliton-pairs with opposite drifting directions ($\Delta\varphi_0$ increases quasi-linearly over time) are present as building blocks (see Supplementary Figure 18).

As shown in Supplementary Figure 14c, the inner spacing τ of the phase-drifting soliton pair exhibits a weak oscillation between 4.7 ps and 5.4 ps, with approximately a sinusoidal oscillation profile which can be approximately described as[27]

$$\tau(z) = \tau_0 + A_\tau \sin(z+\phi_0') \, , \tag{34}$$

with the fitting parameters $\tau_0 = 5.17$ ps, $A_\tau = 0.30$ and $\phi_0' = 0.83\pi$. When we plot the measured results in the interaction plane in which the two orthogonal axis are $\tau\cos(\Delta\varphi_0)$ and $\tau\sin(\Delta\varphi_0)$, as shown in Supplementary Figure 14d we can see that over the consecutive round trips, the phase-drifting soliton pair moved along a circular trajectory whose geometric center has a slight horizontal deviation from the origin.

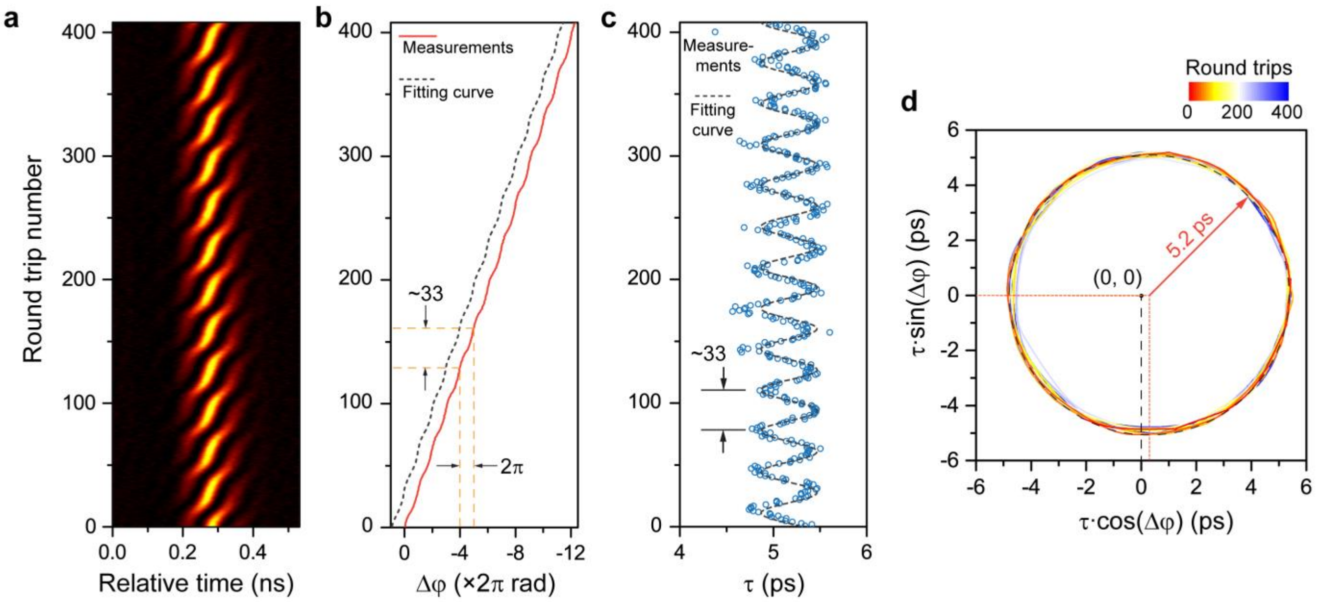

Supplementary Figure 14 | Dynamics of the phase-drifting soliton pair. a, The experimentally acquired DFT signal over 400 consecutive round trips within the time-slot that only contains a phase-drifting soliton pair. **b**, The retrieved phase difference between the two solitons (red solid curve), varied over consecutive cavity round trips. The fitting curve (grey dashed line) is also plotted. **c**, The retrieved inner spacing of the two solitons (blue circle) and the fitting curve (grey dashed line). **d**, The moving trajectory of the phase-drifting soliton pair (plotted in the interaction plane) exhibits a circular trace that is slightly displaced from the origin.

After looking into the internal dynamics of the phase-drifting soliton pair, we now illustrate the macroscopic structure of the soliton supramolecule that contains such soliton pairs. The time-domain trace of the selected time-slots exhibits stable internal spacing between the soliton pair and the single soliton over consecutive cavity round trips (see Supplementary Figure 15a). The entire structure over one cavity round trip is shown in Supplementary Figure 15b, showing the relative positions of the building blocks within different time-slots. As shown in Supplementary Figure 15b, four time-slots are marked by white arrows, within each of which only a phase-drifting soliton pair is trapped. The results indicate that there are long-range forces of repulsion exerted by the single solitons upon the soliton pairs in the units that contained both a single soliton and a soliton pair. These long range forces are apparent in the corresponding optical spectrum recorded by the OSA. As shown in Supplementary Figure 15c, while the drifting phase difference between the two solitons has largely eliminated the fringes on the soliton optical spectrum, local fringes in the vicinity of the Kelly sideband (m = +1 order) are still present. The 0.24 nm fringe period agrees well with the 40 ps internal spacing between the two building blocks. It is also worth mentioning that due to the uncorrelated phase relation between solitons in different time-slots[2], the phase-drifting soliton pairs in different time-slots have the same phase-drifting rates, while their initial phases are observed to be uncorrelated as revealed from the patterns of the DFT signal, shown in Fig. 3g in the main text.

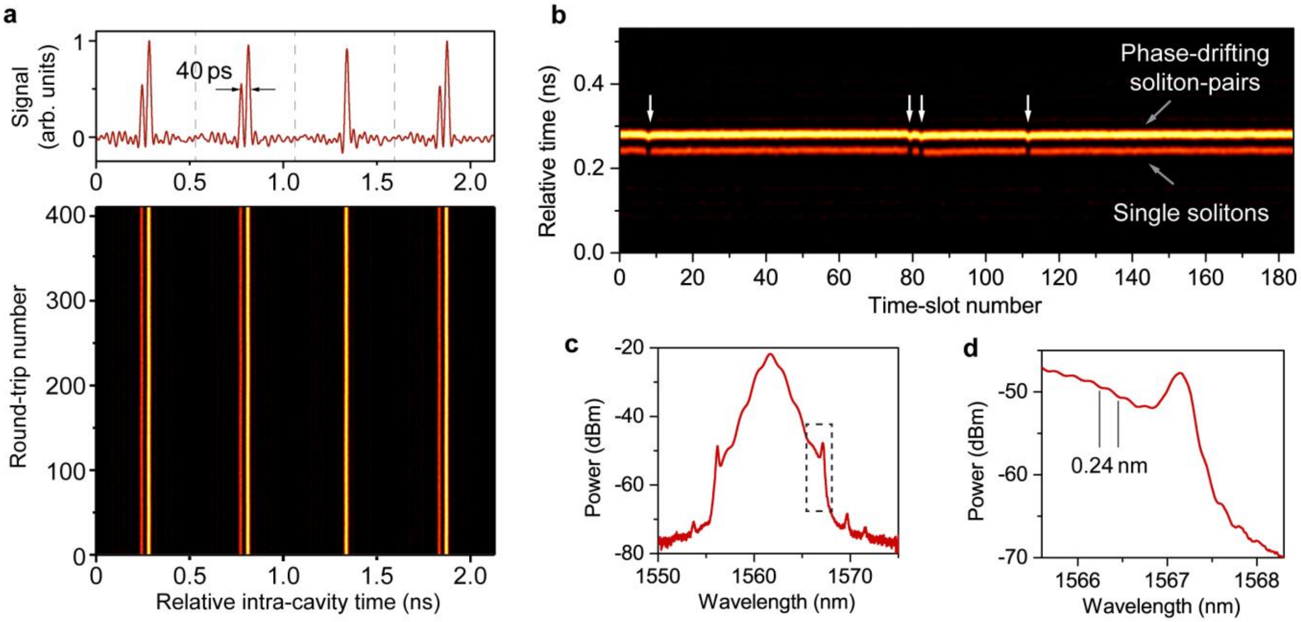

Supplementary Figure 15 | Soliton supramolecules consisting of phase-drifting soliton pairs. a, the time-domain traces of 4 time-slots (out of 184), showing a stable structure over consecutive round trips. **b**, The entire soliton supramolecule consisting 184 time-slots (over one round trip) plotted against time-slot number. **c**, the optical spectrum recorded by the OSA. **d**, Expanded view of the Kelly sideband of $m = +1$ order.

Soliton supramolecules consisting of phase-locked soliton-triplet molecules

In addition to soliton pairs with locked or drifting phase differences, we have also observe larger soliton molecules with three narrowly-spaced solitons (soliton triplets) that appear as fundamental building blocks of soliton supramolecules. We show one example in Fig. 3h in the main text, and the same time-domain trace is plotted in Supplementary Figure 16a. This time-domain trace features optical pulses with three characteristic amplitudes, corresponding to a single soliton, a soliton pair, and a soliton triplet. Within the selected part of the structure shown in Supplementary Figure 16a, several different types of units (corresponding to long-range binding of triplet-triplet, triplet-pair, pair-pair, pair-single, and single-single) can be found within the time-slots. This soliton supramolecular can be stably preserved from round trip to round trip as illustrated in Supplementary Figure 16b. We measure the DFT signal of the soliton supramolecule using the DFT set-up with a 3-km length of dispersive fibre, and we show the result in Supplementary Figure 16c. Due to a relatively large stretch rate of the optical pulse, the DFT signal of different building blocks within one time-slot partially overlap. However the fringes on the DFT signal corresponding to phase-locked soliton pairs and soliton triplets can still be distinguished. Two examples are marked by the arrows in Supplementary Figure 16c. The phase-locked soliton triplets, as revealed from the DFT signal, also have fixed phase differences of π between the three solitons, and the inner-spacing between solitons in these soliton molecules is estimated to be ~6.7 ps. Both the phase differences and inner spacing of these soliton molecules are stably preserved in the soliton supramolecule (see Supplementary Figure 16d).

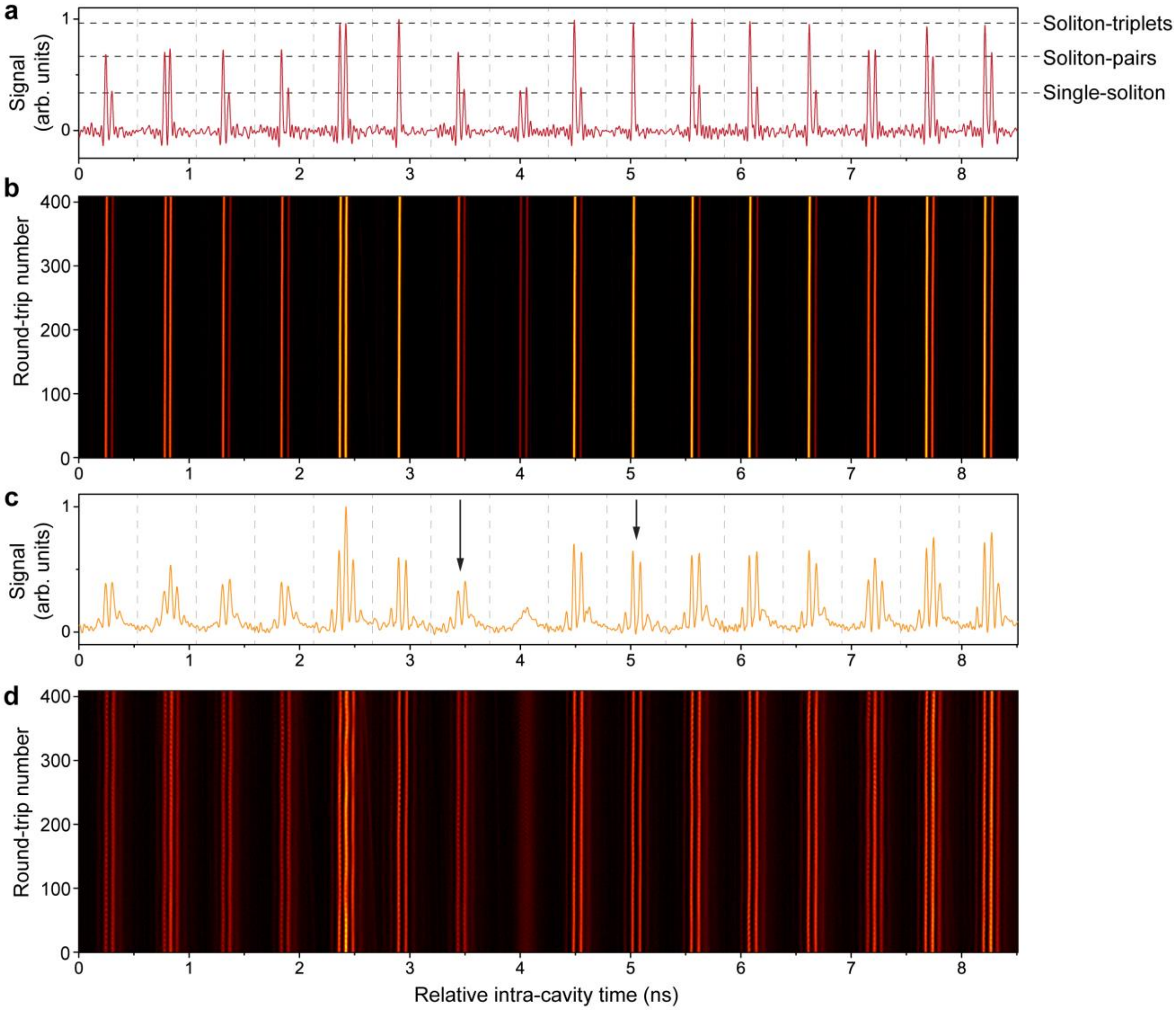

Supplementary Figure 16 | A typical soliton supramolecule including single soliton and phase-locked soliton pairs and triplets. a, The time-domain trace of part of the soliton supramolecule (16 out of 184 time-slots) recorded by the oscilloscope. The three different amplitude levels marked by the black dashed-line corresponds to three different building blocks. **b**, The output signal plotted against round-trip numbers over ~400 cavity round trips. **c**, The DFT signal of the same soliton supramolecule. **d**, The DFT signal plotted against round-trip numbers over ~400 cavity round trips.

Although Supplementary Figure 12 and 16 show constant π phase differences between the solitons, detailed analysis of the DFT signal has revealed that the phase differences are not always constant[28,29], and both the phase differences and inner spacing in these soliton molecules exhibit some random fluctuations[13,27,30]. Nevertheless, the overall supramolecular structure has good long-term stability over many minutes, despite some fluctuation in the parameters of its building blocks.

Coexistence of soliton-pair molecules with different parameters

The examples shown in the main text and the previous Notes of the supplementary information only illustrate the cases that are in the soliton molecules evolved in the same soliton supramolecule share the same parameters (same inner spacing and phase relations). We also occasionally observe that soliton pairs with different parameters could coexist in one soliton supramolecule[28]. We provide two examples to illustrate this additional aspect of structural complexity in the soliton supramolecule. In Supplementary

Figure 17 we show the first example. In Supplementary Figure 17a–c, we show the time-domain trace and the corresponding DFT signal recorded by the oscilloscope as part of this supramolecular structure. Within the time-slots that accommodate soliton pairs, different fringe patterns in the DFT signal are observed (see Supplementary Figure 17b and c), indicating several distinct inner spacing and phase differences in these soliton pairs. The phase differences in most of the soliton pairs slowly drift over consecutive cavity round trips (see Supplementary Figure 17d), while the inner spacing retrieved from the DFT signal features several characteristic values ranging from 4.9 ps to 13 ps (see Supplementary Figure 17e). In the experiments we observe that soliton supramolecules with higher levels of elementary diversity generally exhibit higher noise levels, even though their overall structures are stably preserved in the laser cavity over many minutes.

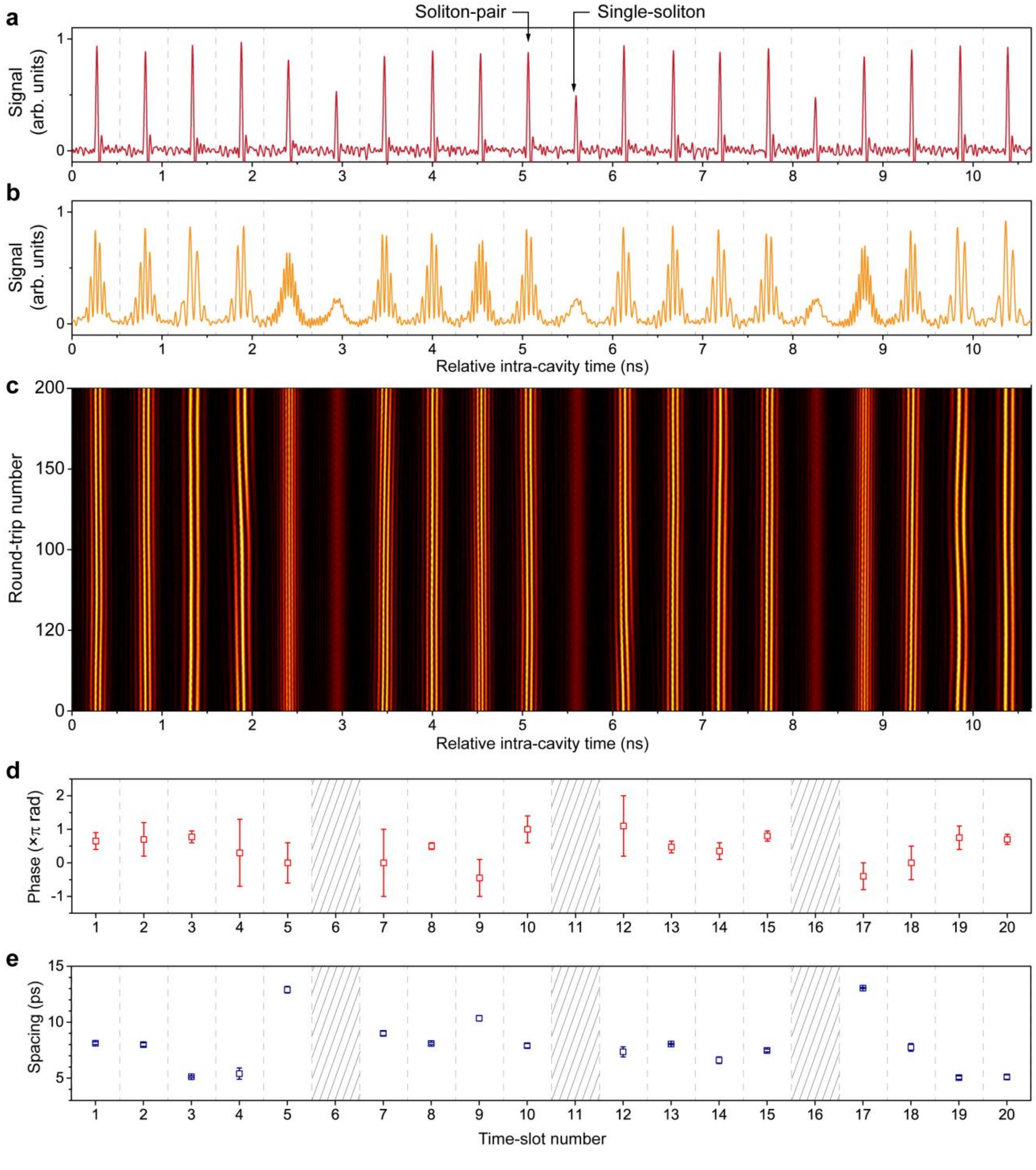

Supplementary Figure 17 | Coexistence of soliton pairs with different inner spacing and phase differences in one supramolecule. a, The time-domain trace of a typical soliton supramolecule in which we plot 20 out of 154 time-slots. **b**, The DFT signal of the supramolecule. **c**, The DFT signal recorded over consecutive 200 cavity round trips, featuring several difference fringe patterns. **d** and **e**, The retrieved phase differences and inner spacing of these soliton pairs. We show the fluctuations of the phase differences and inner spacing with the error bars.

In Supplementary Figure 18 we show the second example in which phase-drifting soliton pairs with different rates of phase drift coexist in the same soliton supramolecule[13,27]. In Supplementary Figure 18a we plot the time-domain trace of the soliton supramolecule, and in Supplementary Figure 18b we plot the corresponding DFT signal. We acquire the DFT signal of this soliton supramolecule over 200 consecutive cavity round trips in the cavity, and the results are plotted against the round-trip number in

Supplementary Figure 18c. The patterns of the DFT traces shown in Supplementary Figure 18c corresponds to different building blocks and are quite different from each other, indicating the co-existence of several types of soliton pairs with different rates of phase drift.

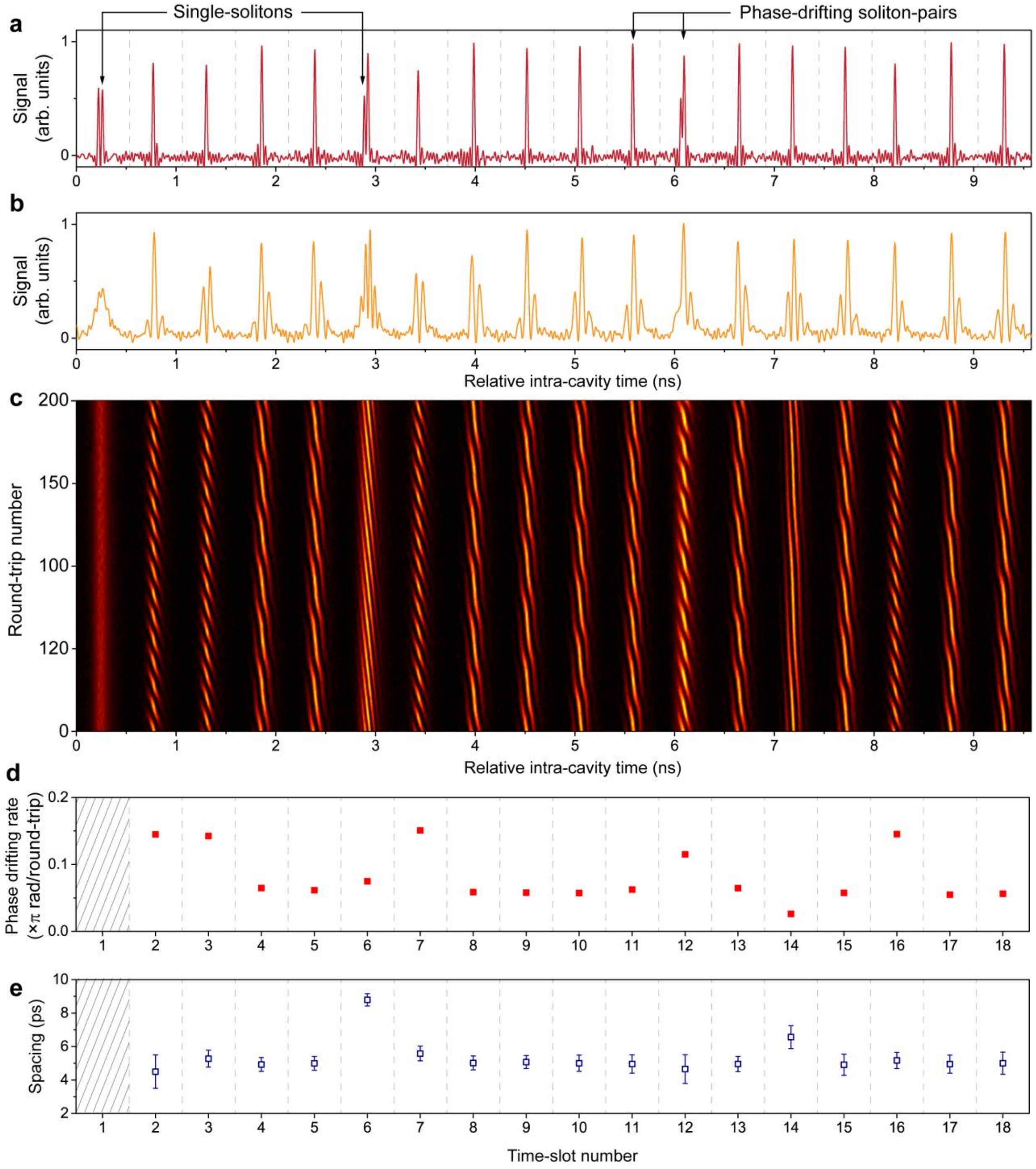

Supplementary Figure 18 | Co-existence of soliton pairs with different inner spacing and phase-drifting rates in the same soliton supramolecule. a, The time-domain trace of the soliton supramolecule in which 18 out of 180 time-slots are plotted. **b**, The corresponding DFT signal. **c**, The DFT signal plotted over consecutive 200 cavity round trips, featuring distinct interferometric fringes. **d** and **e**, The retrieved phase-drift rates and inner spacing for each soliton pair.

Using the equations given in S8.2, we retrieve the rate of phase drift and the inner spacing of these soliton pairs (see Supplementary Figure 18d and e). The results demonstrate that phase-drifting soliton pairs with different parameters can coexist in the same supramolecule with both their drift rates and inner spacings that are at different discrete values[28] (see Supplementary Figure 18d and e). At present, the co-existence of soliton molecules with different parameters in soliton supramolecules has not been comprehensively studied. We note however that this possibility dramatically enriches the potential complexity of the supramolecular structures.

Internal spacing depending on the order of different building blocks

In Supplementary Note 7, we describe three cases in which the different directions of the repulsive forces between solitons that are bound by long-range forces can lead to different configurations of the building blocks within optomechanical lattice units. While in all these three examples the soliton supramolecules contain only single solitons as the building blocks, the example shown in Fig. 3i in the main text illustrates a more complex supramolecular structure. Due to the presence of both single solitons and phase-locked soliton-pairs in this supramolecular structure, several combinations of building blocks (single-single, single-pair, pair-pair) can lead to different internal spacing between the building blocks. This difference in spacing is a probably a consequence of different forces of repulsion that are exerted by a single soliton and a phase-locked soliton pair.

Here we demonstrate that different orders of building blocks in one time-slot of the optomechanical lattice can also lead to different internal spacing. If one Kelly sideband dominates on the laser spectrum, then the direction of the repulsive force is also fixed. Consequently, the order of different building blocks in each time-slot will determine which building block exerts the repulsive force, as, for example, the first soliton described, and which will be perturbed by it, as, for example, the second soliton described in Supplementary Note 7. One example is illustrated in Supplementary Figure 19. In most of the time-slots in this soliton supramolecule shown in Supplementary Figure 19a and b, single solitons appear as the earlier-time building blocks and phase-locked soliton pairs appear as the later ones. Since the $m = -1$ order Kelly sideband at the shorter wavelength is dominant (similar to the case shown in Supplementary Figure 11a–c), the repulsive force in such a unit is mainly exerted by the soliton pair, acting on the single soliton and leading to an internal spacing of ~90 ps (see Supplementary Figure 19a and b). However, in a few time-slots, the order of building blocks is reversed (soliton pair appears as the earlier and soliton pair as the later), leading to the narrower spacing of ~38 ps between the two building blocks (see Supplementary Figure 19a and b). Such non-commutative assembling behavior might be attributed the following mechanism. On one hand, the amplitude of dispersion wave shed from an individual soliton are different from that shed from a soliton pair, leading to different strengths of repulsive forces generated by different solitonic elements. On the other hand, different solitonic elements can respond differently to the same dispersive-wave amplitude, leading to different binding distances. We leave the detailed theoretical analysis as an open question.

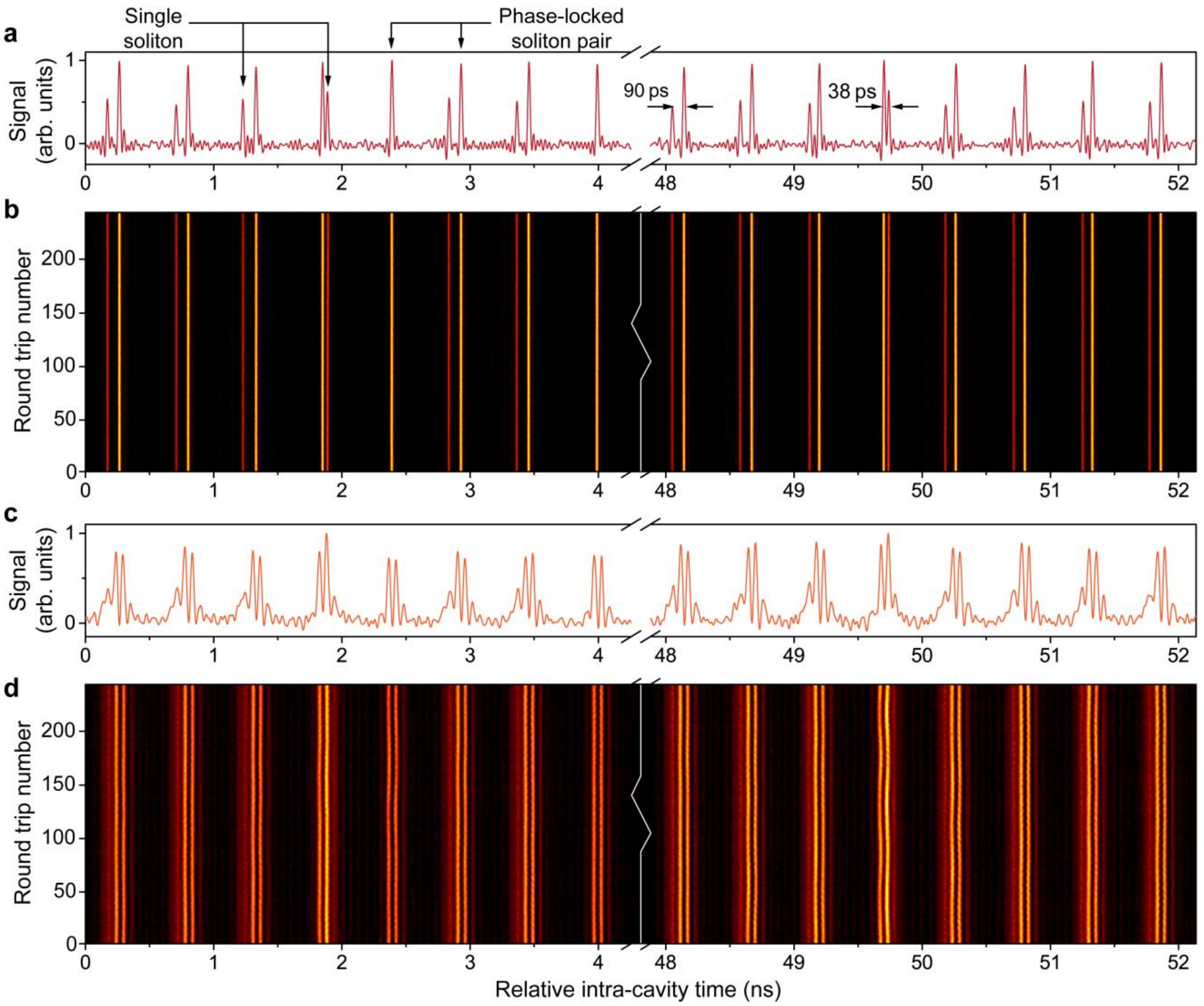

Supplementary Figure 19 | Soliton supramolecule including time-slots in which phase-locked soliton-pairs and single solitons have different orders. a, The time-domain trace of part of the soliton supramolecule (16 out of 154 time-slots) recorded by the oscilloscope. The single-soliton and phase-locked soliton pair in different time-slots can have different orders, leading to different internal spacing. **b**, The output signal plotted against cavity round-trip number (over ~400 round trips). **c**, The corresponding DFT signal of the soliton supramolecule. **d**, The DFT signal plotted against cavity round-trip number.

As illustrated in this Note, the elementary diversity results in multiple levels of complexity in the supramolecular structure, especially when many types of fundamental building blocks, including single solitons and a variety of soliton molecules, comprise the supramolecular assembly.

Supplementary Note 9: Tuning of inter-soliton spacing

Internal spacing tuning of all-double-soliton supramolecule

The soliton-spacing tuning of the all-double-soliton supramolecule can be achieved by tailoring both the intensity of the dominant dispersive wave and amplitude of the acoustic wave in the PCF core. We vary the intensity of the $m = -1$ order Kelly-sideband by adjusting the cavity loss, which is implemented by adjusting the in-cavity tunable attenuator inside the laser cavity (see Supplementary Figure 1). When the total cavity loss is gradually varied from ~6 dB to ~11 dB, we observe that the intensity of the dominant Kelly sideband dropped by a factor of three as shown Fig. 4b in the main text due to gain narrowing. In

this process, the average optical energy from the EDFA decreased by less than 5% due to the highly saturated gain in the EDFA, and this 5% power drop could be compensated by slightly increasing the pump power of the EDFA. The almost unchanged intra-cavity optical energy ensured that the optoacoustic effect in the PCF remained invariant. As illustrated in Fig. 4a in the main text, the increase in the dispersive wave in cavity results in a stronger force of repulsion between the two solitons, leading to larger soliton spacing. In practice, while the basic structure of the ADS supramolecule is stably preserved in the laser cavity, the soliton spacing in the double-soliton unit could be tuned at will within a broad range from 40 ps to 116 ps. The maximum stable internal spacing of an ADS supramolecule that we observe in the experiments is ~170 ps, which approximately equals 260 times the individual soliton duration. This is achieved through increasing further the dispersive wave intensity, which however demanded a higher pump power.

Similarly, the soliton spacing in the triple-soliton unit could also be tuned by tailoring the long-range forces between the solitons. We demonstrate one example of the internal spacing tuning of an ATS supramolecule in Supplementary Figure 20 through adjustment of the intensity of the dispersive wave, similar to the case that we show in Fig. 4 in the main text. As we gradually varied the intensity of the $m = -1$ order dispersive wave (see Supplementary Figure 20a), the spacing between the first and the third soliton in the triple-soliton unit (Δt_{13} in Supplementary Figure 20b) could be tuned within a range from 102 ps to 187 ps, while the spacing between the first and the second soliton (Δt_{12} in Supplementary Figure 20b) could be tuned within a range from 50 ps to 95 ps. Note that the two separations between the consecutive solitons are not necessarily the same in the triple-soliton unit, and during this tuning experiments, the two inter-soliton separations (Δt_{12} and Δt_{23} in Supplementary Figure 20b) actually follow different curves. In Supplementary Figure 20c, we show several time-domain traces recorded by the oscilloscope using the persistence mode.

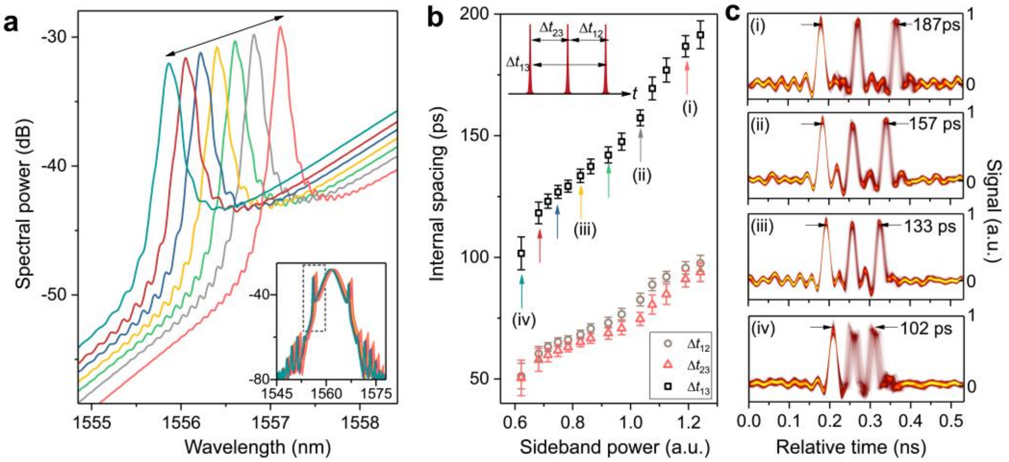

Supplementary Figure 20 | Tuning of the inter-soliton spacing of the all-triple-soliton supramolecule. a, Tailoring of the intensity of the m=–1 order dispersive wave without changing spectral widths of the solitons (see the inset). **b**, The soliton spacing increases as the dispersive wave grows. Quantities Δt_{12}, Δt_{23} and Δt_{13} are defined in the inset. **c**, Four examples of the oscilloscope traces of the ATS supramolecule corresponding to data points marked as (i) – (iv) in **b**.

The soliton spacing could also be tuned by varying the force of attraction, as illustrated in Supplementary Figure 21a. We implement this change by slightly adjusting the cavity length using the intra-cavity delay line (see Supplementary Figure 1). By gradually decreasing the cavity length, the free spectral range (FSR) of the cavity increased, corresponding to a tuning of the lattice frequency of the ADS supramolecule toward the mechanical resonance frequency of the PCF. In our experiment, as the lattice frequency is tuned from 1.8725 GHz to 1.882 GHz (see Supplementary Figure 21b), the soliton spacing in the double-soliton unit decreased from 87 ps to 63 ps corresponding to an increase of the

attractive force as illustrated in Supplementary Figure 21c. In Supplementary Figure 21d we show three exemplary time-domain traces recorded by the oscilloscope with the persistence mode during this tuning process. This tendency changed direction when we increase the lattice frequency above 1.883 GHz, agreeing well with the theoretical predictions described in Supplementary Note 2.

According to Supplementary Equation 13, the repulsive force relies on not only the amplitude of the dispersive wave, but also the exact phase difference ($\Delta\varphi_0$) between the dispersive wave and the perturbed soliton. The examples that we show in Fig. 4 in the main text and Supplementary Figure 21 exhibit nearly invariant phase differences, indicated by the almost unchanged relative positions of the spectral fringes upon the dominant Kelly sideband. However, during the repetition tuning experiment shown in Supplementary Figure 21, we observe a slight shift of $\Delta\varphi_0$ around 1.883 GHz. In some other tuning experiments, we find that the variation of the phase difference between the dispersive wave and the perturbed soliton could be quite significant during the tuning of spacing.

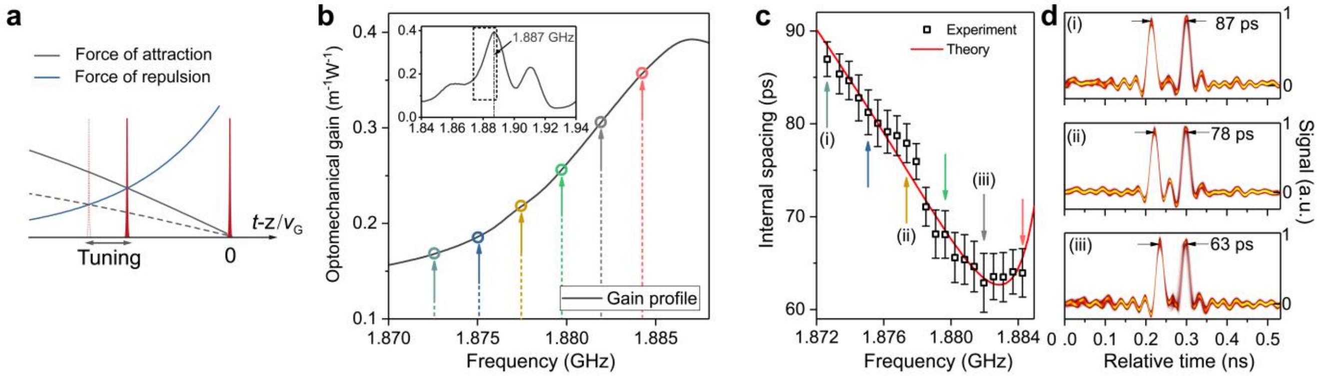

Supplementary Figure 21 | Soliton spacing tuning of the ADS supramolecule by tailoring the amplitude of the acoustic wave in the PCF core. a, Tailoring the double-soliton spacing by varying the force of attraction. **b**, Tuning the lattice frequency of the ADS supramolecule by varying the laser cavity length. The optomechanical gain band of the solid-core PCF is plotted in the inset, showing a mechanical resonance at 1.887 GHz. **c**, The soliton spacing first decreases as the lattice frequency of the supramolecule is tuned toward the mechanical resonance frequency, and then increases at frequencies above 1.882 GHz. The soliton spacing could be tuned from 63 ps to 87 ps. The experimental results are plotted as black squares, with the theory curve plotted in red. **d**, We show three exemplary oscilloscope traces on the left panels corresponding to the data points marked as (i)–(iii).

To obtain the soliton spacing, we average many recorded data, while the standard deviations are shown as the error bars in Fig. 4c in the main text and Supplementary Figures 20c and 21c, which is obtained using all the double-soliton units in the supramolecular structures. We observe a slightly higher spacing jitter, with a maximum jitter value of ~10 ps, near the upper and lower edges of the spacing-tuning range as shown in Fig. 4d in the main text and Supplementary Figure 20d. We always optimized our system near the midpoint of the tuning range, leading to the large spacing jitter observed at the edge of the tuning rage. Nevertheless, if we maintained all the system parameters, we did not observe any degradation of the pulse timing jitter over many minutes.

Supplementary Note 10: Self-assembling dynamics

Experimental details for adding and removing solitons

By strongly perturbing the laser pump power, we are able to add or remove solitons to or from the existing soliton supramolecules. In this section, we provide experimental details. To add solitons, as shown in Fig. 5a in the main text, we modulate the pump power to generate an abrupt increase by ~15%. The

voltage change (1.5 V) from the electrical pulse generator has a 5-ns rising edge. However, the laser diode has longer response time, leading to a relatively-slow (typical ~ 1 µs) change of the laser pump power. This modulation of the laser pump power has two consequences. First, all the existing solitons experience a sudden decrease of their group velocities due to the increased intensity, leading to a longer cavity round-trip time and therefore the observed bending of the yellow lines after the pump power increases. Second, the noise background of the laser arose due to the pump-power increase, and a few solitons are then generated from noise light spikes[31]. These newly-generated solitons could be captured by the soliton supramolecule, increasing the soliton numbers in some units, as shown in the expanded view of the transient process in Fig. 5b, marked by the white arrow in Fig. 5a in the main text. The entire supramolecular structure, nevertheless, remained stable during the transient process, and the birth of new solitons is the result of complex gain- and nonlinearity-related processes in the laser cavity[31].

We can also remove some individual solitons from a soliton supramolecule without destroying its overall supramolecular structure. One typical result is shown in Fig. 5c in the main text. When the pump power is decreased by ~10% over a trailing edge of a few µs, many of the double-soliton units degraded into single-soliton units in which one of the solitons in these units diminished over a few hundred round trips in the cavity. The expanded view of the transient process highlighted by the white arrow in Fig. 5c is shown in Fig. 5d in the main text. The underlying mechanism of this soliton moving process could possibly be interpreted in the following way: First, when the pump power is decreased, the peak intensities of laser solitons are reduced and the solitons acquired higher group velocities. Some solitons trapped in the time-slots would tend to escape from their equilibrium positions due to the perturbation. The net frequency-shift experienced by these solitons would then lead to lower gain in the EDFA, since the carrier frequencies of these solitons deviated from the gain maximum. As a result, the group velocities of these solitons in this unit would become even higher due to the lower intensity, leading to a faster rate of escape (see Fig. 5d in the main text). This positive feedback accelerates the process of escape, and finally the solitons disappeared in the background. In the experiments, the change of pump power actually affected every soliton in the supramolecule in the same way, since the response time of the EDFA is much longer than the cavity round-trip time. However, due to laser noise perturbations, an abrupt change of laser pump power pushes some “unlucky” solitons outside the trapping potential, which means that these solitons happened to be slightly more vulnerable than the others. They might happen to slightly deviate a bit more from their equilibrium positions, or they might happen to have slightly lower intensities than the others. By contrast, when the laser operates stably, a perturbation from laser noise will not typically lead to soliton escape due to the presence of the trapping potential. Once the abrupt change of the laser pump power is strong enough, the stability of some “unlucky” solitons is ultimately destroyed, leading to the disappearance of these solitons over a few hundred round trips in the cavity. The other solitons in the supramolecule, however, successfully adjusted their energies and group velocities, transforming to the new working point of the laser, and forming a stable supramolecular structure with a new pattern.

A significant difference between the processes of adding and removing solitons lies in the durations of these processes. While the soliton removal process starts immediately after the drop in the laser pump power (see Fig. 5a in the main text), we observe that the process of soliton addition required a longer preparation stage after the laser pump power is increased of about 8000 roundtrips as shown in Fig. 5c in the main text. This difference in time scale is due to a difference in physical process. The newly-generated solitons are born from the laser noise background, and the birth of new laser solitons generally results from complicated dissipative and nonlinear processes whose durations are observed to be several hundred cavity round trips[31].

Self-adjustment of the soliton positions in soliton supramolecules

In the experiments we observe that the newly-generated solitons would occasionally appear away from the equilibrium positions within the time-slots of a supramolecular structure. Despite this initial timing offset, due to the presence of the long-range forces, these solitons are quickly dragged backed to the balanced positions, leading to a smooth transient process from one supramolecular pattern to another. In Supplementary Figure 22 we show one example of the self-adjustment process. In the time-slots marked by the white arrows, the newly-generated solitons did not appear at the equilibrium positions of the supramolecular structure. Over several thousands subsequent cavity round trips, these solitons adjusted themselves by moving to the equilibrium positions and eventually became the new building blocks of the soliton supramolecule.

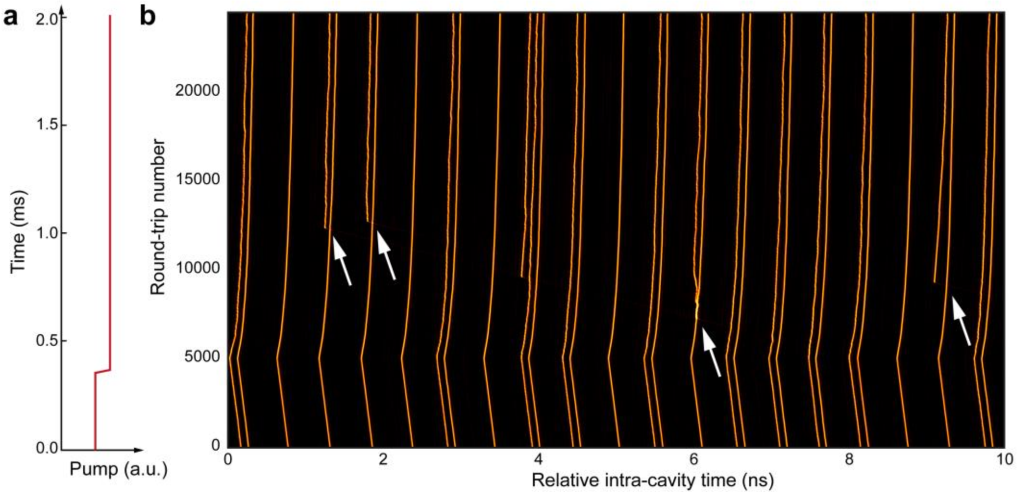

Supplementary Figure 22 | Self-adjustment of internal spacing in the soliton supramolecule. a, An abrupt increase of pump power by ~15% with a ~ 1 μs rising edge. **b,** Some solitons are born from the background with initial positions away from the balanced positions (or trapping potential centers) of the supramolecular structure, as marked by the white arrows. However, over a few thousands of cavity round trips, these newly-generated solitons would automatically move to the equilibrium positions due to the presence of the long-range forces in the supramolecule.

The self-adjustment of soliton positions could occasionally follow more complicated trajectories in the experiments. One example is shown in Supplementary Figure 23, in which a newly-generated soliton happened to appear between two long-range-bound solitons in one time-slot (see Supplementary Figure 23b, marked by the white arrow). The transient process show that the initial second soliton is then slowly pushed away due to the long-range forces between these solitons and become the third soliton in this time-slot, while the newly-generated soliton took over the position of the initial second soliton. This self-adjustment process roughly took a few thousands of cavity round trips before the new triple-soliton unit became stable.

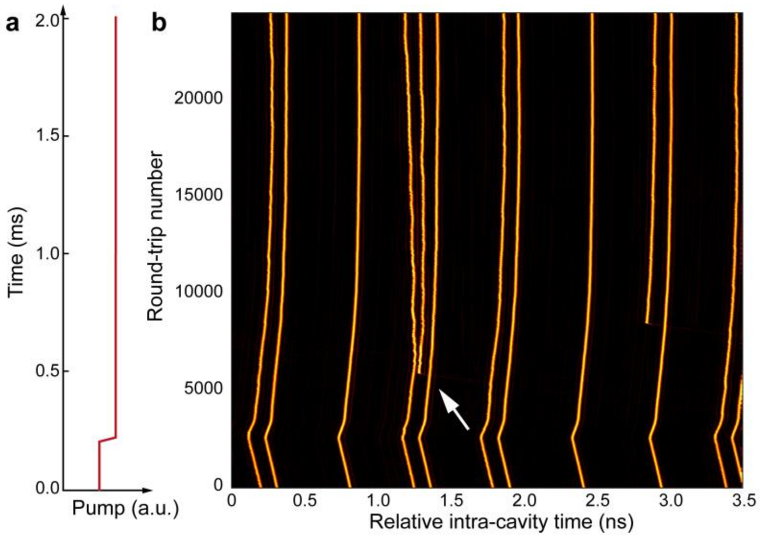

Supplementary Figure 23 | Another example of the self-adjustment of internal spacing in the supramolecular structure. a, An abrupt increase of pump power by ~15% with a few-μs rising edge. **b**, A new soliton is generated between the two solitons in a double-soliton unit (marked by the white arrow). The spacing between the solitons in this unit self-adjusted over the following thousands of cavity round trips, leading to the formation of a stable triple-soliton unit.

The self-adjustment dynamics of the internal spacing in the supramolecular structure is also observed during the process of soliton removal. When the intermediate soliton is removed from one triple or quadruple soliton unit of a supramolecule, the neighboring solitons slowly drift toward the empty position in the trapping potential. We show two examples in Supplementary Figure 24. When the pump power is decreased abruptly by ~ 15%, the quadruple-soliton unit marked by the white arrow (see Supplementary Figure 24b) lost its third soliton, and the fourth soliton slowly drifted and occupied the empty position, leading to a degradation of the quadruple-soliton down to a triple-soliton unit. In the same soliton supramolecule, a triple-soliton unit in another time-slot lost its second soliton, and the third soliton took over the position, forming a stable double-soliton unit as shown in Supplementary Figure 24c.

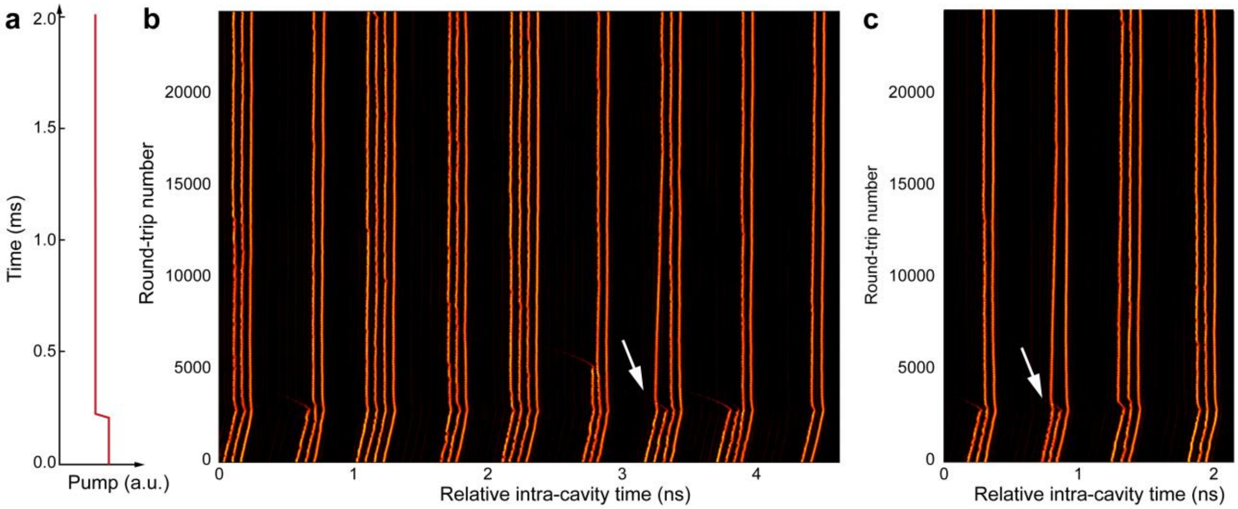

Supplementary Figure 24 | The self-adjustment of internal spacing in a soliton supramolecule during the soliton-removing process. a, An abrupt decrease of pump power by ~15% with a few μs rising edge. **b**, The quadruple-soliton unit lost its third soliton due to a decrease of the laser pump power, after which the internal spacing between solitons in this unit self-adjusted, forming a stable triple-soliton unit. **c**, In another time-slot, a triple-soliton unit lost its second soliton, and a stable double-soliton unit formed.

Soliton transfer between different time-slots.

In this section we describe an interesting dynamic process that we observe in the experiments in which individual solitons could be transferred between different time-slots in a soliton supramolecule by using a carefully induced perturbation. Two examples are illustrated in Supplementary Figure 25. In the first example the laser pump power is modulated so that it has an abrupt dip of ~ 50 μs, as shown in Supplementary Figure 25a. We observe that this modulation in laser pump power resulted in the escape of the second-soliton of a double-soliton unit from its original time-slot, which is then re-captured in another time-slot where a double-soliton unit is trapped. This re-capture process resulted in the upgrading of this double-soliton unit into a triple-soliton one as we show in Supplementary Figure 25b and c. During the transfer process, the energy of the soliton significantly decreased (see Supplementary Figure 25c). However the soliton did not completely disappear and eventually re-gained its amplitude due to the recovery of the laser pump power. This process can be regarded as a combination of the soliton removal and addition processes. By increasing the width of dip in the pump power modulation, a longer transition distance has also be observed in the experiments. As shown in Supplementary Figure 25d–f, the escaped solitons could drift over four time-slots before being re-captured again in the supramolecular structure. This transferred soliton is also observed to be heavily attenuated during the transient process, but is still able to recover its original profile and is then trapped within another time-slot of the supramolecule. These experimental results provide one more possibility for controlling the fine structure of the soliton supramolecule in which communication between different time-slots occurs.

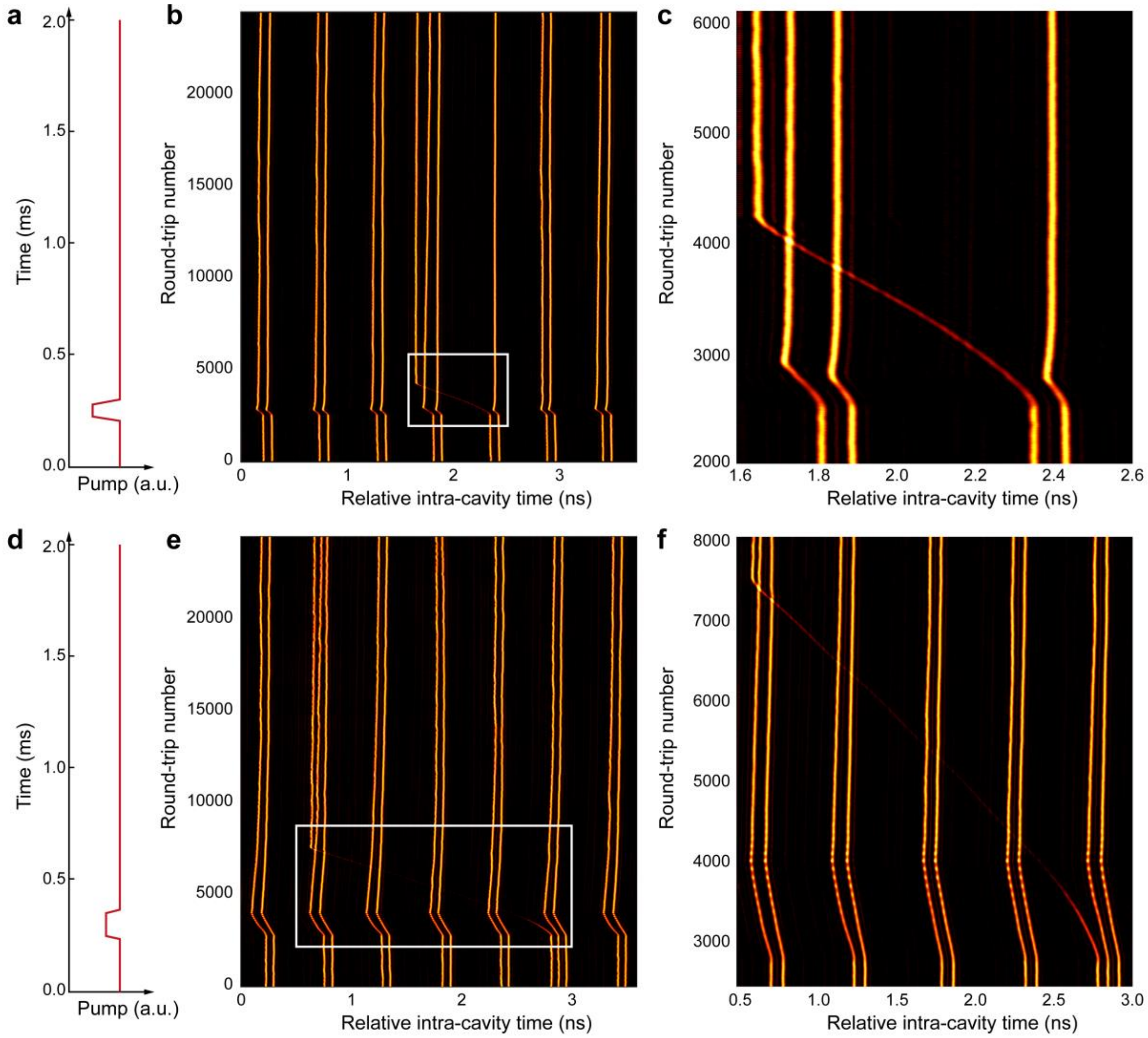

Supplementary Figure 25 | Two examples of soliton transfer between different time-slots in the supramolecule. **a**, An abrupt dip of the pump power by ~25% over a duration of 50 μs. **b**, One soliton of a double-soliton unit escaped from its original position and is later captured within another double-soliton unit, upgrading this unit into a triple-soliton unit. **c**, Expanded view of the region marked by the white box in **b**. **d**, A wider dip of pump power by ~12% over 100 μs. **e**. One soliton in a triple-soliton unit consequently escaped its initial position, moved across four time-slots and is eventually captured within a double-soliton unit. **f**, Expanded view of the region during the soliton-transfer process.

Supplementary References

1. Pang, M. et al. Stable subpicosecond soliton fiber laser passively mode-locked by gigahertz acoustic resonance in photonic crystal fiber core. *Optica* **2,** 339–342 (2015).
2. He, W., Pang, M. & Russell, P. St.J. Wideband-tunable soliton fiber laser mode-locked at 1.88 GHz by optoacoustic interactions in solid-core PCF. *Opt. Express* **23,** 24945–24954 (2015).
3. He, W., Pang, M., Menyuk, C. R. & Russell, St.J. P. Sub-100-fs 1.87 GHz mode-locked fiber laser using stretched-soliton effects. *Optica* **3,** 1366–1372 (2016).
4. Pang, M., He, W., Jiang, X. & Russell, P. St.J. All-optical bit storage in a fibre laser by optomechanically bound states of solitons. *Nat. Photon.* **10,** 454–458 (2016).
5. Kang, M. S., Nazarkin, A., Brenn, A. & Russell, P. St. J. Tightly trapped acoustic phonons in photonic crystal fibres as highly nonlinear artificial Raman oscillators. *Nat. Phys.* **5,** 276–280 (2009).

6. Haus, H. A. Mode-locking of lasers. *IEEE J. Sel. Top. Quantum Electron.* **6,** 1173–1185 (2000).
7. Hofer, M., Fermann, M. E., Haberl, F., Ober, M. H. & Schmidt, A. J. Mode locking with cross-phase and self-phase modulation. *Opt. Lett.* **16,** 502–504 (1991).
8. Nelson, L. E., Jones, D. J., Tamura, K., Haus, H. A. & Ippen, E. P. Ultrashort-pulse fiber ring lasers. *Appl. Phys. B* **65,** 277–294 (1997).
9. Tamura, K., Nelson, L. E., Haus, H. A. & Ippen, E. P. Soliton versus nonsoliton operation of fiber ring lasers. *Appl. Phys. Lett.* **64,** 149–151 (1994).
10. Malomed, B. A. Bound solitons in the nonlinear Schrödinger-Ginzburg-Landau equation. *Phys. Rev. A* **44,** 6954–6957 (1991).
11. Akhmediev, N. N., Ankiewicz, A. & Soto-Crespo, J. M. Multisoliton Solutions of the Complex Ginzburg-Landau Equation. *Phys. Rev. Lett.* **79,** 4047–4051 (1997).
12. Stratmann, M., Pagel, T. & Mitschke, F. Experimental Observation of Temporal Soliton Molecules. *Phys. Rev. Lett.* **95,** 143902 (2005).
13. Herink, G., Kurtz, F., Jalali, B., Solli, D. R. & Ropers, C. Real-time spectral interferometry probes the internal dynamics of femtosecond soliton molecules. *Science* **356,** 50–54 (2017).
14. Pilipetskii, A. N., Golovchenko, E. A. & Menyuk, C. R. Acoustic effect in passively mode-locked fiber ring lasers. *Opt. Lett.* **20,** 907–909 (1995).
15. Bondeson, A., Lisak, M. & Anderson, D. Soliton perturbations: a variational principle for the parameters. *Phys. Scr.* **20,** 479–485 (1979).
16. Gordon, J. P. Dispersive perturbations of solitons of the nonlinear Schrödinger equation. *J. Opt. Soc. Am. B* **9,** 91–97 (1992).
17. Noske, D. U., Pandit, N. & Taylor, J. R. Source of spectral and temporal instability in soliton fiber lasers. *Opt. Lett.* **17,** 1515–1517 (1992).
18. Kelly, S. M. J. Characteristic sideband instability of periodically amplified average soliton. *Electron. Lett.* **28,** 806–808 (1992).
19. Dennis, M. L. & Duling III, I. N. Experimental study of sideband generation in femtosecond fiber lasers. *IEEE J. Quantum Electron.* **30,** 1469–1477 (1994).
20. Becker, P., Olsson, A. & Simpson, J. *Erbium-Doped Fiber Amplifiers: Fundamentals and Technology*. (Academic Press, 1991).
21. Agrawal, G. P. *Nonlinear Fiber Optics*. (Academic Press, 2007).
22. Haus, H. A. & Mecozzi, A. Noise of mode-locked lasers. *IEEE J. Quantum Electron.* **29,** 983–996 (1993).
23. Kodama, Y. & Hasegawa, A. Generation of asymptotically stable optical solitons and suppression of the Gordon–Haus effect. *Opt. Lett.* **17,** 31–33 (1992).
24. Zwanzig, R. *Nonequilibrium Statistical Mechanics*. (Oxford University Press, 2001).
25. Arnold, L. *Stochastic differential equations*. (Wiley, 1974).
26. Rohrmann, P., Hause, A. & Mitschke, F. Two-soliton and three-soliton molecules in optical fibers. *Phys. Rev. A* **87,** 1–10 (2013).
27. Krupa, K., Nithyanandan, K., Andral, U., Tchofo-Dinda, P. & Grelu, P. Real-Time Observation of Internal Motion within Ultrafast Dissipative Optical Soliton Molecules. *Phys. Rev. Lett.* **118,** 243901 (2017).
28. Soto-Crespo, J. M., Akhmediev, N., Grelu, P. & Belhache, F. Quantized separations of phase-locked soliton pairs in fiber lasers. *Opt. Lett.* **28,** 1757–1759 (2003).
29. Peng, J. & Zeng, H. Build-Up of Dissipative Optical Soliton Molecules via Diverse Soliton Interactions. *Laser Photon. Rev.* **12,** 1800009 (2018).
30. Shi, H., Song, Y., Wang, C., Zhao, L. & Hu, M. Observation of subfemtosecond fluctuations of the pulse separation in a soliton molecule. *Opt. Lett.* **43,** 1623–1626 (2018).
31. Herink, G., Jalali, B., Ropers, C. & Solli, D. R. Resolving the build-up of femtosecond mode-locking with single-shot spectroscopy at 90-MHz frame rate. *Nat. Photon.* **10,** 321–326 (2016).
